# Suppression of BMP-7 by histone deacetylase 2 promoted apoptosis of renal tubular epithelial cells in acute kidney injury

**DOI:** 10.1038/cddis.2017.552

**Published:** 2017-10-26

**Authors:** Taotao Ma, Cheng Huang, Qingqing Xu, Yang Yang, Yaru Liu, Xiaoming Meng, Jun Li, Min Ye, Hong Liang

**Affiliations:** 1 The Key Laboratory of Major Autoimmune Diseases, Anhui Province, Anhui Institute of Innovative Drug, School of Pharmacy, Anhui Medical University, No. 81 Meishan Road, Hefei 230032, Anhui, China; 2 State Key Laboratory of Natural and Biomimetic Drugs, School of Pharmaceutical Sciences, Peking University, 38 Xueyuan Road, Beijing 100191, China

## Abstract

Cisplatin, a highly effective and widely used chemotherapeutic agent, has a major limitation for its nephrotoxicity. Currently, there are no therapies available to treat or prevent cisplatin nephrotoxicity. We recently identified a novel strategy for attenuating its nephrotoxicity in chemotherapy by histone deacetylase (HDAC) inhibitors via epigenetic modification to enhance bone morphogenetic protein 7 (BMP-7) expression. Cisplatin upregulated the activity of HDAC2 in the kidney. Inhibition of HDAC with clinically used trichostatin A (TSA) or valproic acid (VPA) suppressed cisplatin-induced kidney injury and epithelial cell apoptosis. Overexpression of HDAC2 promotes CP-treated tubular epithelium cells apoptosis. Chromatin immunoprecipitation assay clearly detected HDAC2 assosiation with BMP-7 promoter. Western blot and immunofluorescence results demonstrated that the expression of BMP-7 was clearly induced by TSA or VPA *in vivo* and *in vitro*. Interestingly, administration of recombinant BMP-7 (rhBMP-7) reduced cisplatin-induced kidney dysfunction. Moreover, BMP-7 treatment suppressed epithelial cell apoptosis and small interfering RNA-based knockdown of BMP-7 expression abolished HDAC inhibitors suppression of epithelial cell apoptosis *in vitro*. Results of current study indicated that TSA or VPA inhibited apoptosis of renal tubular epithelial cells via promoting the level of BMP-7 epigenetically through targeting HDAC2. Hence, HDAC inhibitors could be useful therapeutic agents for the prevention of cisplatin nephrotoxicity.

Cisplatin (CP), which directly interferences with DNA synthesis and apoptosis, is usually used to treat numerous types of cancers. However, nephrotoxicity, a major side effect of chemotherapy, limits its use. Therefore, it is necessary to find an effective measure to attenuate nephrotoxicity induced by CP. Currently, limited understandings of the cellular mechanisms of acute kidney injury (AKI) complicate the development of an effective treatment.^1^ Abundant evidence shows that apoptotic cell death is a prominent and characteristic feature of AKI caused by nephrotoxic medications.^3^ Thus, improved mechanistic understanding of renal cell apoptosis has generated new therapeutic targets in AKI.

Available evidence suggest histone deacetylase (HDAC) enzymes have a close correlation with cell proliferation and apoptosis.^4^ It is suggested that HDAC enzymes balance the acetylation activities of histone acetyltransferases on chromatin remodeling and play essential roles in regulating gene transcription.^5^ Up to now, 18 mammalian HDACs have been identified and divided into four classes (classes I, II, III and IV), which depend on phylogenetic analysis and sequence similarity to yeast factors, classes I, II and IV HDACs require zinc ions as cofactors, whereas class III HDACs are NAD+-dependent enzymes known as sirtuins (SIRT1-7). In general, class I HDACs (HDAC1, 2, 3 and 8), characterized by a highly conserved deacetylase domain which is flanked by short amino- and carboxy-terminal extensions, are ubiquitously expressed and exert a strong catalytic effect on histone lysine residues, predominantly localizing in the nuclear compartment of the cell.^6^ In the kidney, emerging evidence demonstrate that HDACs contribute to renal damage. Although histone acetylation/deacetylation of AKI is a nascent field, the available information has already provided compelling evidence that chromatin biology plays a critical role in this disease.^7, 8^

HDAC inhibitors are now used to treat different types of cancer. However, recent studies suggest that inhibitors of the Zn^2+^-dependent HDACs, including hydroxamic acids, electrophilic ketones, cyclic peptides, short-chain fatty acids and benzamides, also have immunomodulatory activity and antiapoptotic effects in kidney disease.^4, 9, 10, 11^ The emerging role of HDAC inhibitor with phenylthiobutanoic acids has been reported to enhance renal recovery and attenuate renal fibrosis.^12, 13^ It is suggested that the potential roles of HDAC inhibitors may be responsible for promoting renal regeneration and functional recovery in AKI induced by CP. The aim of this study was to determine whether HDAC inhibitors could suppress cisplatin nephrotoxicity, and if so the underlying mechanisms would be determined. Our results demonstrated that administration of TSA or VPA suppressed renal tubular epithelial cell apoptosis. Furthermore, the protective activities of HDAC inhibitors were mediated through upregulation of a novel antiapoptotic protein called BMP-7 in renal tubular epithelial cells. The study revealed that opposing regulation of BMP-7 contributed to the role of HDAC2 on renal tubular epithelial cell apoptosis.

## Results

### Cisplatin induced apoptosis of renal tubular epithelial cell *in vivo* and *in vitro*

**Figure 1:**
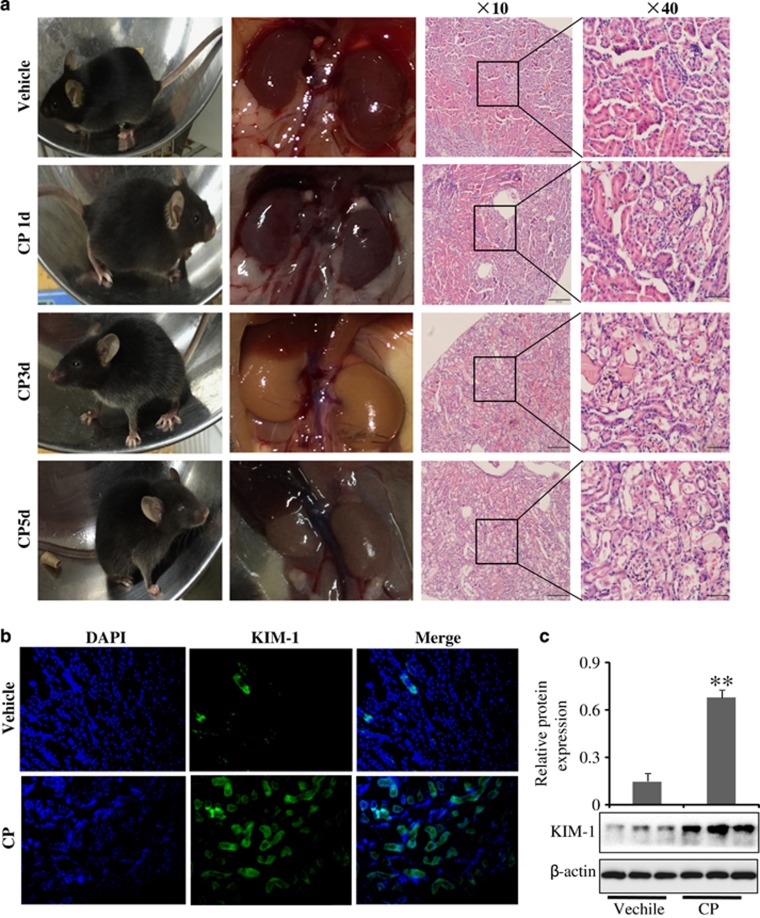
Differential levels of kidney dysfunction in response to cisplatin treatment. (**a**) Representative macroscopic kidney, mice appearances and HE staining at different time after CP treatment. (**b**) Immunofluorescence analysis of KIM-1 protein in mice induced by CP. Data were represented as mean±S.D. of 10 animals of each group. (**c**) Western blot analysis of KIM-1 protein treated in mice induced by CP. Data were represented as mean±S.D. of three independent experiments. **P<0.01 versus vehicle group

To determine renal response to cisplatin treatment, kidney tissues were harvested at 1, 3 and 5 days after cisplatin administration. It was found that mice fur were messy on the third day of CP injection, and their movements were slow. Macroscopic kidneys appeared whitening as CP induced significant lesions in mice (Figure 1a). Furthermore, the serum creatinine (Cr) and blood urea nitrogen (BUN) levels were significantly increased in kidney (Supplementary Figure S1), suggesting CP could successfully induced AKI mice model on the third day.

Kidney injury molecule-1 (KIM-1) is a biomarker of kidney injury.^14^ Expression of KIM-1 determined by immunofluorescence staining and western blot revealed that KIM-1 protein expression was clearly upregulated in the CP-treated group on the third day compared with the vehicle (Figures 1b and c). As expected, similar results were also found in human kidney epithelial cells (HK-2) and mouse kidney epithelial cells (mTEC) (Supplementary Figure S2).

**Figure 2:**
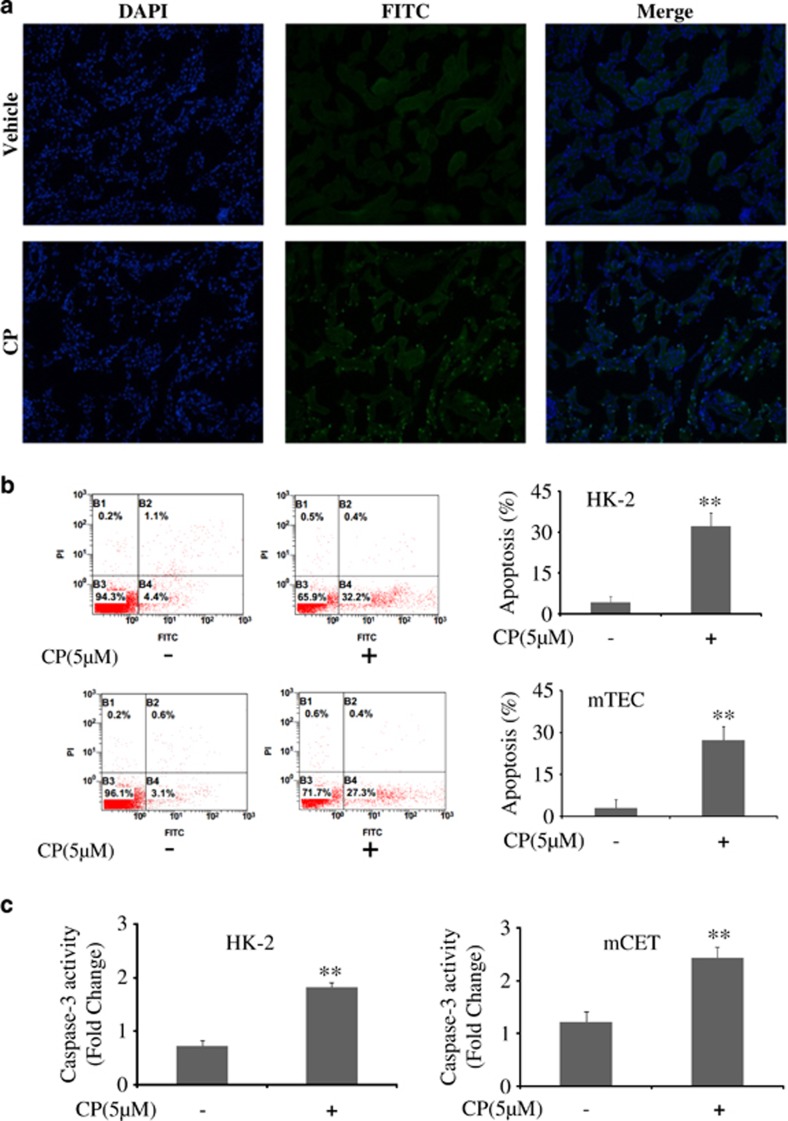
Cisplatin induced apoptosis of renal tubular epithelial cell *in vivo* and *in vitro*. (**a**) Representative images of TUNEL staining in different groups. Scale bars show 200*μ*m. Data were represented as mean±S.D. of 10 animals of each group. (**b**) Flow cytometry analysis of apoptosis in CP-treated HK-2 and mTEC cells. (**c**) The activity of caspase-3 was quantified in CP-treated HK-2 and mTEC cells according to the Materials and Methods section. Data were represented as mean±S.D. of three independent experiments. **P<0.01 versus control group

Then *in vivo* and *in vitro* studies were used to monitor apoptosis induced by CP. As shown in Figures 2a and b, cisplatin administration significantly increased renal tubular epithelial cell apoptosis in the kidney.

Bcl-2 family members act as anti- or pro-apoptotic regulators that are involved in a wide variety of cellular activities. Apoptosis regulator Bax, also known as bcl-2-like protein 4, is a member of the Bcl-2 gene family.^15^ The protein level of Bax was increased while Bcl-2 was decreased with CP treatment in both HK-2 and mTEC cells (Supplementary Figure S3).

As caspase-3 plays a central role in the execution phase of cell apoptosis, the activity of caspase-3 in HK-2 and mTEC cells was both detected. Interestingly, as shown in Figure 2c, CP induced a large increase in caspase-3 activity.

### HDAC inhibitors suppressed cisplatin-induced kidney dysfunction

**Figure 3:**
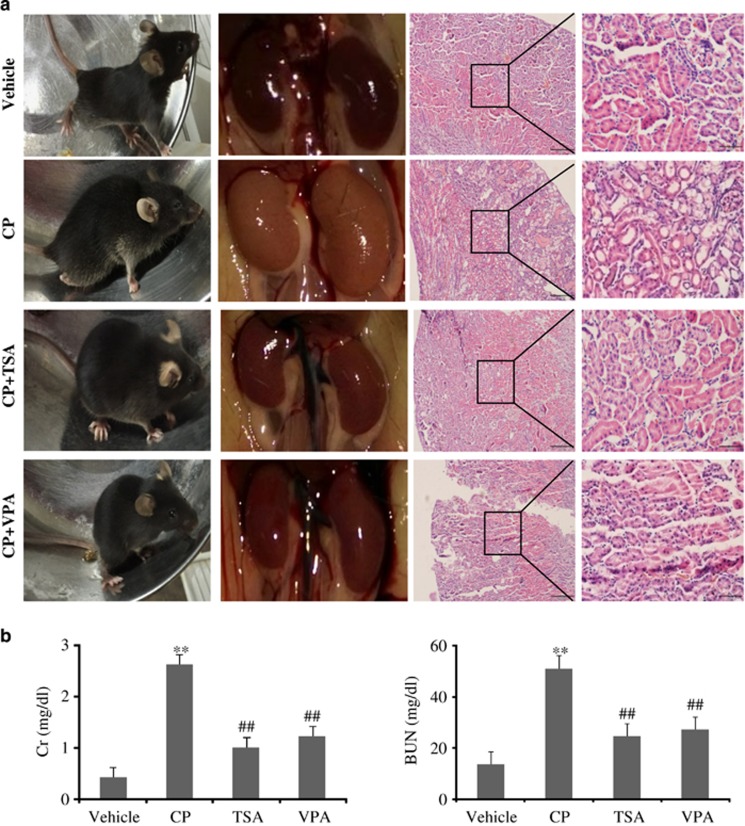
HDAC inhibitor TSA and VPA administration suppressed cisplatin-induced kidney dysfunction. (**a**) Representative macroscopic appearances of the kidneys and HE staining. Magnification: × 10 and × 40. (**b**) Serum Cr and BUN levels were quantified after various treatments in mice. Serum Cr and BUN were indicators of the kidney function Data were represented as mean±S.D. of 10 animals of each group. **P<0.01 *versus* vehicle group; ^##^*P*<0.01 *versus* CP-induced group

To determine the effects of HDAC inhibitors on HDAC-mediated cisplatin-induced nephrotoxicity, TSA or VPA was administered. Macroscopic appearances showed CP induced significant lesions that produced whitening of the kidneys in mice while TSA administration largely suppressed kidney dysfunction. Consistent with improved kidney function with TSA administration, hematoxylin and eosin (H&E)-stained renal tissues appeared to shows less tubular necrosis, cast formation and preservation of a brush border in the cisplatin+TSA administered group as compared with the cisplatin-treated group (Figure 3a). In addition, the increased BUN and Cr levels were also significantly attenuated with TSA (Figure 3b). These results indicated that TSA had a protective effect on CP-induced AKI.

Administration of HDAC I-specific inhibitor, VPA, also significantly improved the kidney function, which was associated with better preservation of kidney morphology, suggesting VPA is capable of suppressing cisplatin-induced kidney injury (Figure 3).

**Figure 4:**
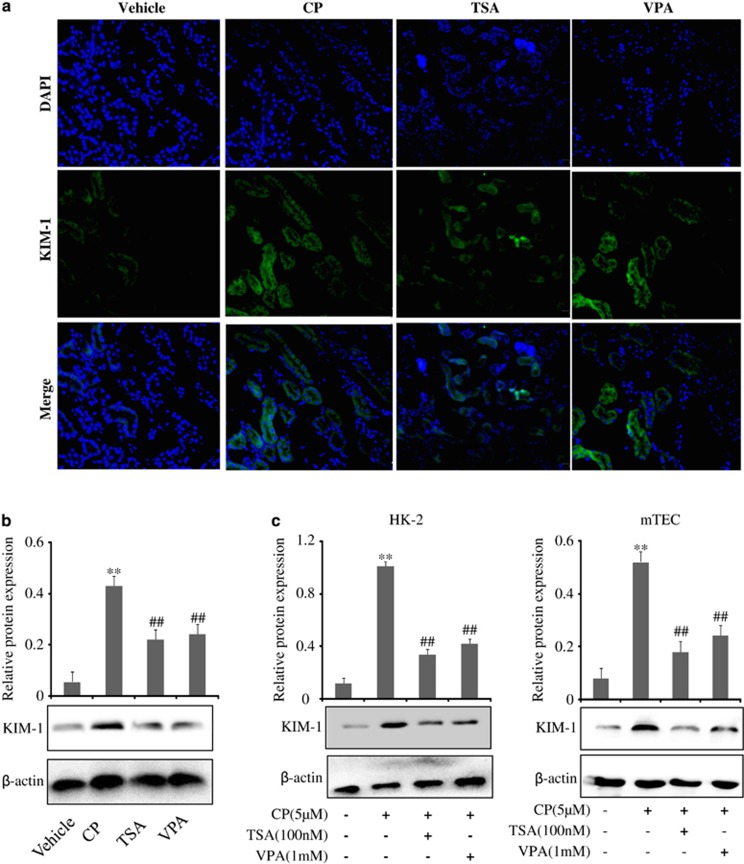
Effects of TSA and VPA on expression of KIM-1 *in vivo and in vitro*. (**a**) Immunofluorescence analysis of KIM-1 protein treated with TSA and VPA. (**b**) Western blot analysis on the expression of KIM-1 in the CP-induced AKI in the presence of TSA or VPA. Data were represented as mean±S.D. of three independent experiments. **P<0.01 *versus* vehicle group, ^##^*P*<0.01 *versus* CP-treated group. (**c**) Effects of TSA and VPA on expressions of KIM-1 induced by CP in HK-2 cells and mTEC cells. Data were represented as mean±S.D. of three experiments. **P<0.01 *versus* control group, ^##^*P*<0.01 *versus* CP alone

Immunofluorescence staining and western blot analysis revealed that KIM-1 protein expression was clearly upregulated in the CP-treated group compared with the vehicle group while TSA or VPA could decrease the expression of KIM-1 protein in Figures 4a and b. Consistent with *in vivo* data, KIM-1 expression in a cisplatin-induced renal tubular epithelial cell was also decreased by TSA or VPA treatment (Figure 4c).

### HDAC inhibitors suppressed cisplatin-induced renal tubular epithelial cell apoptosis

**Figure 5:**
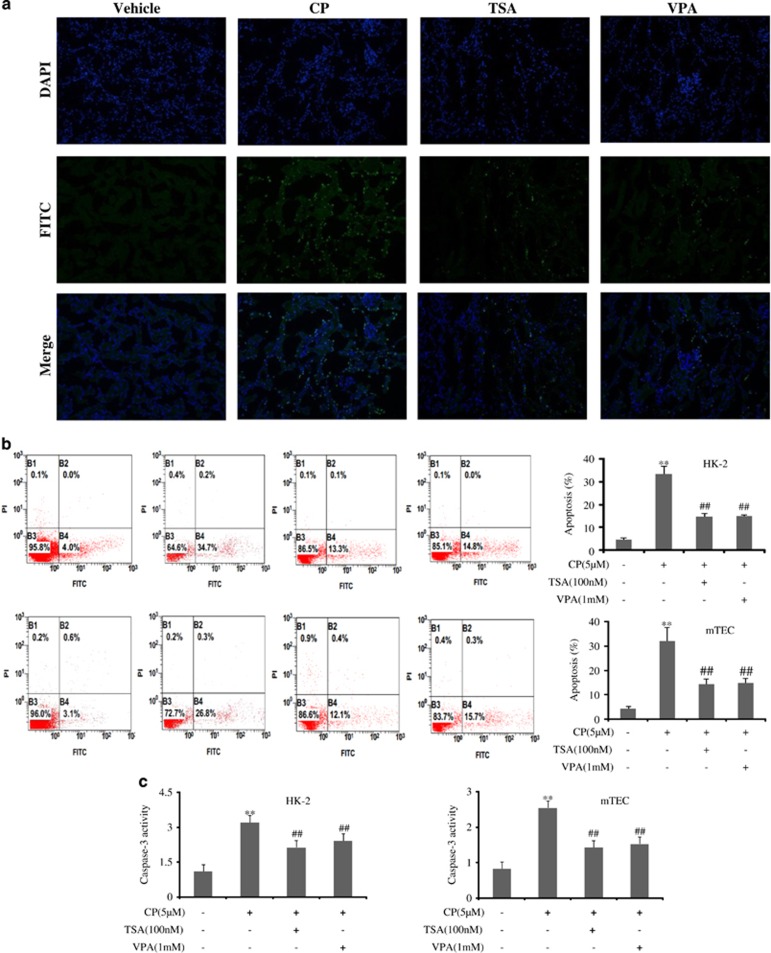
HDAC inhibitors suppressed cisplatin-induced renal tubular epithelial cell apoptosis. (**a**) Representative images of TUNEL staining in different groups. Scale bars show 200*μ*m. Data were represented as mean±S.D. of 10 animals of each group. (**b**) Apoptosis of HK-2 and mTEC cells treatment with TSA or VPA analyzed by flow cytometry. (**c**) Effects of TSA or VPA on the activity of caspase-3 in HK-2 and mTEC cells. The activity of caspase-3 was quantified according to the Materials and Methods section. Data were represented as mean±S.D. of three independent experiments. **P<0.01 *versus* control group, ^##^*P*<0.01 *versus* CP alone

To investigate the anti-apoptotic effect of TSA or VPA, TUNEL stain was used. Numerous TUNEL-positive cells were observed in CP-treated AKI in contrast with the vehicle group, while administration of TSA or VPA could significantly decrease TUNEL-positive cells (Figure 5a). Consistent with *in vivo* study, apoptosis assay by flow cytometric analysis was carried out in HK-2 and mTEC cell lines, and the results indicated that TSA or VPA showed high activity against apoptosis with CP treatment (Figure 5b).

The protein levels of Bcl-2 and Bax were also detected by western blot analysis. It was demonstrated that HDAC inhibitor, TSA or VPA, was associated with an increase in Bcl-2 and a decrease in Bax protein expression induced by CP in HK-2 and mTEC cells (Supplementary Figure S4). Interestingly, the activity of caspase-3 inhibited by TSA or VPA was also detected (Figure 5c).

### Overexpression of HDAC2 promoted CP-treated tubular epithelium cells apoptosis

**Figure 6:**
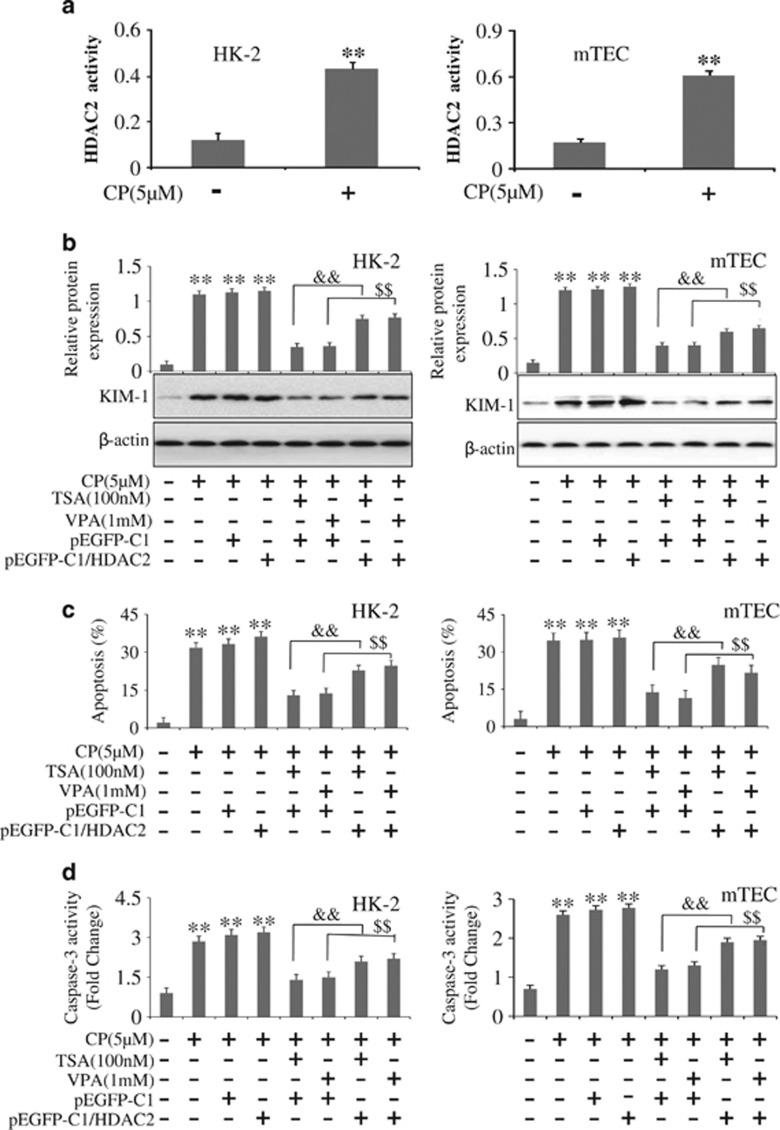
Overexpression of HDAC2 promotes CP-treated tubular epithelium cells apoptosis. (**a**) CP regulated the activity of HDAC2 in HK-2 and mTEC cells. (**b**) Effects of overexpression of HDAC2 on expression of KIM-1 in HK-2 and mTEC cells analyzed by western blot. (**c**) Effects of overexpression of HDAC2 on apoptosis in HK-2 and mTEC cells analyzed by flow cytometry. (**d**) Effects of overexpression of HDAC2 on activities of Caspase-3 in HK-2 and mTEC cells. The activity of caspase-3 was quantified according to the Materials and Methods section. Data were represented as mean±S.D. of three independent experiments. **P<0.01 *versus* control group, ^&&^*P*<0.01 versus TSA+pEGFP-C1; ^$$^*P*<0.01 versus VPA+pEGFP-C1

Available evidence suggested that HDACs were critically involved in kidney diseases. To determine which isoforms of HDACs were induced in response to cisplatin treatment, reverse transcriptase-PCR was used to detect expression of HDACs in HK-2 and mTEC cells harvested at 24 h after cisplatin administration. It was shown that cisplatin induced a large increase in HDAC2 expression, whereas a moderate increase was seen for the expressions of HDAC1 (Supplementary Figure S5). In order to inspect CP influence on HDAC2 activity, deacetylase activity was measured by a commercial colorimetric HDAC2 assay kit. It was demonstrated that CP treatment induced a significantly increase in HDAC2 activity (Figure 6a).

To gain insight into the potential function of HDAC2 in AKI, overexpression of HDAC2 by transfecting them with pEGFP-C1-HDAC2 was used in CP-treated HK-2 and mTEC cells (Supplementary Figure S6). Compared with transfected with a control plasmid, overexpression of HDAC2 with addition of TSA or VPA significantly decreased KIM-1 expression (Figure 6b). To confirm the role of HDAC2 in CP-induced apoptosis, the flow cytometric analysis was used. Similar results were observed. Overexpression of HDAC2 with addition of TSA or VPA significantly increased apoptotic cells in contrast with a control plasmid (Figure 6c). The apoptosis assay by flow cytometric analysis is shown in Supplementary Figure S7.

As shown in Supplementary Figure S8, overexpression of HDAC2 was also associated with a decrease in Bcl-2 and an increase in Bax protein expression compared with transfected with control plasmid. Furthermore, the activity of caspase-3 was significantly upregulated in CP-treated HK-2 and mTEC cells transfected with pEGFP-C1/HDAC2 with the addition of TSA or VPA (Figure 6d). Hence, HDAC2 may mainly function by promoting apoptosis of HK-2 and mTEC cells in AKI.

### HDAC inhibitors upregulated BMP-7 expression in AKI

**Figure 7:**
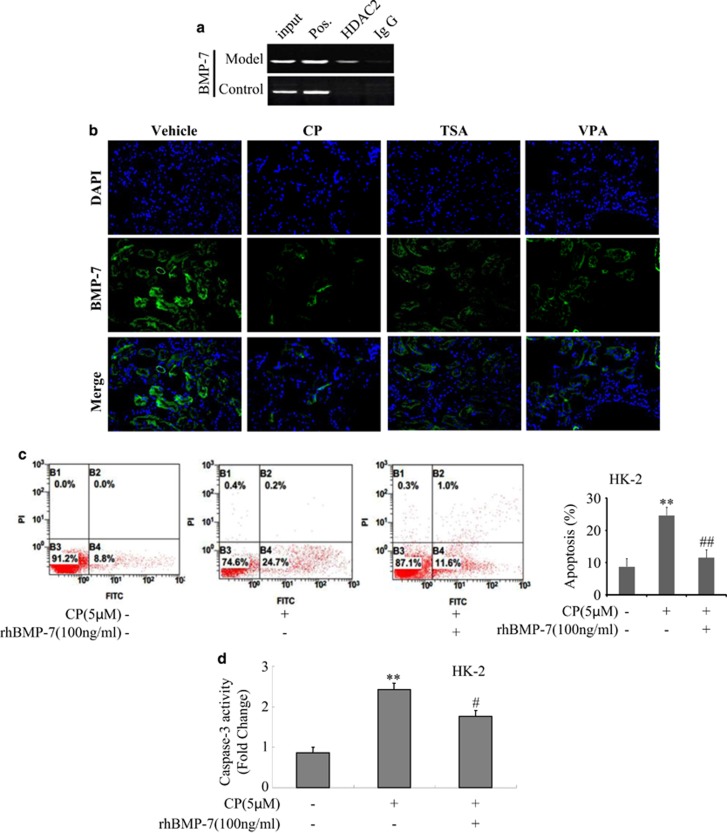
HDAC inhibitor downregulated BMP-7 expression in mice with AKI *in vivo* and *in vitro*. (**a**) HDAC2 binding site is identified in BMP-7 promoter region by chip assay. (**b**) Immunofluorescence analysis of BMP-7 protein treated with TSA or VPA *in vivo*. Data were represented as mean±S.D. of 10 animals of each group. (**c**) Apoptosis of HK-2 and mTEC cells treatment with rhBMP-7 analyzed by flow cytometry in HK-2 cells. (**d**) The activity of caspase-3 was quantified in CP-treated HK-2 in the presence of rhBMP-7 according to Materials and Methods section. Data were represented as mean±S.D. of three independent experiments. **P<0.01 *versus* control group, ^#^*P*<0.05, ^##^*P*<0.01 *versus* NC

To determine the molecular mechanism of HDAC2 in AKI, chromatin immunoprecipitation (ChIP) assay was used. As shown in Figure 7a, ChIP assay clearly detected HDAC2 was recruited to the promoter region of BMP-7 gene. Surprisingly, HDAC inhibitor, TSA or VPA treatment partially but significantly promoted the level of BMP-7 *in vivo* and *in vitro*. Localization studies demonstrated that CP treatment suppressed BMP-7 expression in tubular epithelium cells, and a more pronounced increase was observed in the presence of TSA or VPA (Figure 7b). Consistent with *in vivo* studies,*in vitro* data were also confirmed in HK-2 and mTEC cells by western blot. Inhibitors of HDAC induced a large increase in BMP-7 expression in HK-2 and mTEC cells (Supplementary Figure S9).

To clarify whether HDAC2 is responsible for the regulation of BMP-7, small interfering RNA (siRNA)-based knockdown HDAC2 was constructed into HK-2 and mTEC cells (Supplementary Figure S10). Surprisingly, downregulation of HDAC2 by siRNA significantly increased the level of BMP-7 (Supplementary Figure S11).

Over the last decade BMP-7 has emerged as a critical renal protective protein that safeguards the kidney against a variety of stimuli that caused renal injury.^17, 18^

To determine whether BMP-7 was effective in preventing cisplatin-induced kidney injury, HK-2 cells was treated with recombinant BMP-7. As expected, cisplatin-induced epithelial cell apoptosis was significantly reduced in the presence of BMP-7 by flow cytometric analysis (Figure 7c).

Furthermore, as shown in Supplementary Figure S12, rhBMP-7 significantly decreased KIM-1 and Bax protein expressions while increased Bcl-2 protein level, suggesting that cells apoptosis was suppressed by BMP-7. Similarly the activity of caspase-3 was significantly downregulated in CP-treated HK-2 cells with BMP-7 (Figure 7d). These results indicated BMP-7 might mainly function by suppressing apoptosis in AKI.

### HDAC inhibitors suppressed CP-induced epithelial cell apoptosis through BMP-7

**Figure 8:**
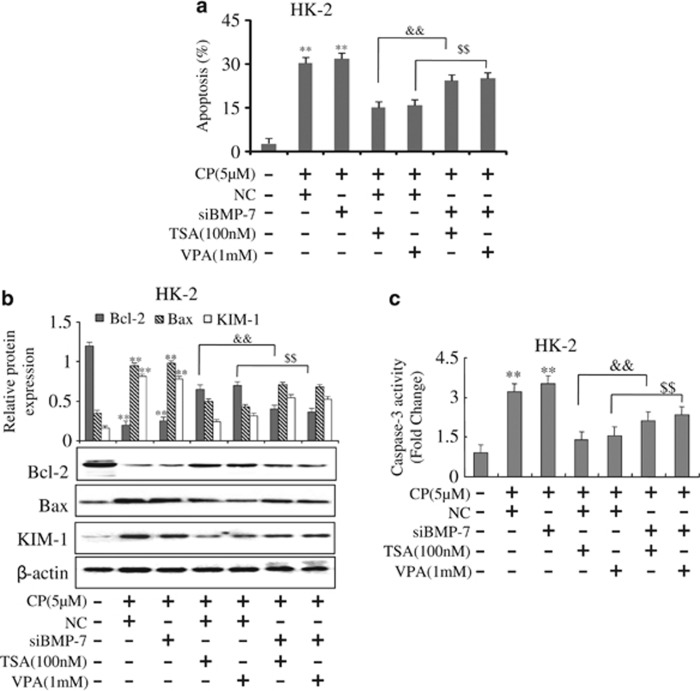
Effects of HDAC inhibitors on apoptosis in BMP-7 knockdown cells. (**a**) Apoptosis was analyzed by flow cytometry in BMP-7 knockdown HK-2 cells. (**b**) Effects of TSA or VPA on expressions of Bcl-2 and Bax induced by CP in BMP-7 knockdown HK-2 cells analyzed by western blot. (**c**) Effects of TSA or VPA on the activity of caspase-3 induced by CP in BMP-7 knockdown HK-2 cells. The activity of caspase-3 was quantified according to the Materials and Methods section. Data were represented as mean±S.D. of three independent experiments. **P<0.01 *versus* control group; ^&&^*P*<0.01 versus TSA+NC; ^$$^*P*<0.01 versus VPA+NC

To further confirm that BMP-7 was the mediator of TSA or VPA-mediated suppression of cisplatin-induced apoptosis, siRNA-based knockdown BMP-7 was constructed into HK-2 cells (Supplementary Figure S13). Flow cytometric analysis indicated that the numbers of apoptosis renal tubular epithelial cells transfected with siBMP-7 were largely increased, which was induced by CP; unexpectedly, it could not be inhibited when treating with TSA or VPA (Figure 8a). Similar findings were shown by western blot that the protein level of Bax and the activity of caspase-3 were increased in cells transfected with siBMP-7 while the level of Bcl-2 protein was decreased. TSA or VPA -mediated suppression of cisplatin-induced epithelial cell apoptosis was abolished with BMP-7 knockdown (Figures 8b and c). Taken together, HDAC inhibitor, TSA or VPA, suppressed CP-induced epithelial cell apoptosis through BMP-7.

## Discussion

Emerging evidence has demonstrated CP is an effective chemotherapeutic agent used to treat a wide variety of solid tumors since 1978. However, nephrotoxicity is the major clinical side effect of CP, and there are no treatments available to prevent it. Therefore, it is necessary to find a new therapy basing on the mechanism of CP-induced nephrotoxicity. It is well accepted that renal tubular epithelial cells are the major sites of cell injury and apoptosis is the common histopathological feature during cisplatin nephrotoxicity.^19^ Available evidence suggests that the major apoptotic pathway in cisplatin nephrotoxicity is the intrinsic or mitochondrial pathway. The anti-apoptotic gene, Bcl-2, and the pro-apoptotic gene, Bax, play key roles in cisplatin-induced AKI.^20^ Encouragingly, Jiang *et al.*^21^ reported that knockout of Bax diminished renal tubular epithelial cells apoptosis during cisplatin nephrotoxicity.

HDACs are known to have an important role in cellular physiology and gene regulation. Despite significant similarities in a large family of HDAC enzymes, different sets of HDAC isoenzymes are probably relevant for the regulation of different target genes or proteins. HDAC inhibitors are being considered as anticancer drugs because of their potential to induce differentiation or apoptosis preferentially in cancer cells. The hydroxamic acids such as TSA and suberoylanilide hydroxamic (SAHA), which are isolated from bacterial and fungal sources, are firstly noted for their abilities to induce cell cycle arrest and cancer cell differentiation. It is later appreciated that these effects are more broad-spectrum inhibitors capable of inhibiting class I and II HDACs, while VPA, the short-chain fatty acids inhibitor, which is relatively weak and display some class I specificity, inhibited the catalytic activity of HDACs, preferentially of class I, presumably by binding to the catalytic center of the enzyme. A recent study by Khan’s group^4^ found that VPA improved beta-cell proliferation and function as well as reduced beta-cell apoptosis through HDAC inhibition. Similarly, Chiu *et al.*^10^ reported curcumin targeting HDAC prevented apoptosis and improved motor deficits in Park 7 (DJ-1)-knockout rat model of Parkinson's disease. Moreover, Brochier *et al.*^11^ provided a molecular understanding of the specific outcomes of HDAC inhibition and suggested that strategies aimed at enhancing p53 acetylation might be therapeutically viable for capturing the beneficial effects in the CNS. These findings that HDAC inhibitors can suppress apoptosis in non-cancer cells also suggest distinct physiological contexts between cancer and non-cancer cells.

In the current study, the number of TUNEL-positive cells increased significantly after CP injection whereas HDAC inhibitor, TSA or VPA, could reduce the number of cells positive for TUNEL staining. Furthermore, the expression of pro-apoptotic Bax was increased by CP treatment while TSA or VPA significantly suppressed the level of Bax and caspase-3. Conversely, the expression of anti-apoptotic Bcl-2 was significantly increased after TSA or VPA treatment. Our studies showed for the first time that HDAC inhibitors, TSA and VPA, were capable of suppressing renal tubular epithelial cell apoptosis through modulation expression of Bcl-2 family proteins and activity of caspase-3.

As one of the members of the HDAC family, HDAC2 has been shown to play a potential role in organ development and cellular homeostasis by regulating gene expressions.^22^ Available evidence suggests that HDAC2 is critically involved in kidney diseases. Noh’s group^23^ reported HDAC2 was a key regulator of diabetes and transforming growth factor-beta1 (TGF-*β*1)-induced renal injury. Recent studies reported that decreasing HDAC2 expression level was in parallel with increasing acetyl histone H3 and associated with the renoprotective effect in sepsis-induced AKI.^24^ In this current research, the activity of HDAC2 significantly was increased in the CP treatment group. Overexpressed HDAC2 in CP-treated HK-2 and mTEC cells by transfecting them with pEGFP-C1-HDAC2 significantly suppressed the anti-apoptotic effects of TSA or VPA. Our finding that HDAC inhibitors could suppress apoptosis in renal tubular epithelial cells also suggested distinct physiological contexts between cancer and AKI.

Recent studies reported that HDAC-dependent repression of bone morphogenetic protein family transcription was a critical event during the pathogenesis of disease.^13^ Wang *et al.*^26^ demonstrated that HDAC2 could recruit BMP-7 promoter in mammary epithelial cells. Furthermore, Hsing *et al.*^24^ also found that dexmedetomidine protected against septic AKI through inhibiting HDAC2 and increasing BMP-7. It is now well accepted that BMP-7, a key member in the TGF-*β* superfamily, plays an important role in kidney diseases.^27^ Zeisberg’s group^28^ reported treatment with rhBMP-7 led to improved renal function, histology and survival in mice deficient in the alpha3-chain of type IV collagen and MRL/MpJlpr/lpr lupus mice. Similarly, Tampe’s study showed administration of BMP-7 could successful inhibit experimental kidney fibrosis.^29^ Kamiura’s group^30^ reported treatment with rhBMP-7 rescued the cisplatin-induced apoptosis in renal tubular epithelial cells from MyoR^−/−^mice, and Yu’s group^31^ also found BMP-7 could attenuate TGF-*β*1-induced apoptosis in NRK-52E cells. Consistent with these observations, BMP-7 protects renal tubular epithelial cells from apoptosis in kidney diseases. This indicated kidney injury induced by CP might be modulated by the activation of HDAC, perhaps through the reactivation of BMP-7.

With regard to the underlying cellular mechanisms, our current study found that HDAC2 recruited to the promoter region of BMP-7 *gene* and HDAC inhibitor, TSA or VPA, could promote the level of BMP-7 *in vivo* and *in vitro*. Furthermore siRNA-based knockdown HDAC2 significantly upregulated the level of BMP-7. In addition, administration of rhBMP-7 reduced cisplatin-induced kidney dysfunction. Moreover, BMP-7 treatment suppressed epithelial cell apoptosis and siRNA-based knockdown of BMP-7 expression abolished HDAC inhibitors suppression of epithelial cell apoptosis *in vitro*. Taken together, this study provided novel evidence for a nephro-protective role of HDAC inhibitors in CP-induced AKI. Interestingly, HDAC2-stimulated apoptosis of renal tubular epithelial cells was at least in part mediated by BMP-7. Thus, HDAC inhibitors could be useful therapeutic agents for the prevention of cisplatin nephrotoxicity.

## Materials and methods

### Material reagents

CP, TSA, dimethyl sulfoxide, VPA, MTT (3-(4,5-dimethylthiazol-2-yl)-2,5-diphenyltetrazoliumbromide) were obtained from Sigma-Aldrich (St. Louis, MO, USA). BMP-7, KIM-1, Bcl-2 and Bax antibodies were purchased from Santa Cruz (Santa Cruz biotechnology, Dallas, TX, USA). *β*-Actin antibody and secondary antibodies for goat anti-rabbit IgG HRP and rabbit anti-goat IgG HRP were purchased from Bioworld Technology (Nanjing, China). Cr and BUN assay kit were purchased from Siemens (Siemens Healthcare Diagnostics, Tarytown, NY, USA).

### Animals and drug administration

C57BL/6 mice supplied by the Experimental Animal Center of Anhui Medical University were used to establish the AKI model. All the animal experiments were performed in accordance with the Regulations of the Experimental Animal Administration issued by the State Committee of Science and Technology of China. Efforts were made to minimize the number of animals used and their suffering. Animals were maintained in accordance with the Guides of Center for Developmental Biology, Anhui Medical University for the Care and Use of Laboratory. Animals and all experiments used protocols approved by the institutions’ subcommittees on animal care. The mice were randomly divided into four groups (*n*=10 per group): vehicle group, model group, TSA treatment group and VPA treatment group. Cisplatin was dissolved in saline at a concentration of 1 mg/ml. Mice were given a single intraperitoneal injection of either saline or cisplatin (20 mg/kg body weight). Some of these animals received TSA (1mg/kg body weight) via gavage or VPA (1 mg/kg body weight) or vehicle every 24 h starting 2 days before cisplatin administration. Animals were killed 72 h after cisplatin injection, and blood and kidney tissues were collected. Kidney tissues were processed for histology, TUNEL assay, protein and RNA isolation.

### Cell culture

HK-2 and mTEC, kindly provided by Prof. Huiyao Lan, were cultured in DMEM/F-12 (HyClone, Logan, UT, USA) supplemented with 10% (vol/vol) heat-inactivated fetal bovine serum (Merck Millipore, Darmstadt, Germany) at 37 °C in a humidified incubator under 5% CO_2_. Cells were treated with cisplatin (5 *μ*mol/l, 24h) with/without 0.1 *μ*g/ml of BMP-7 for 24 h and then harvested for RNA isolation or used to determine apoptosis. To determine CP regulation of BMP-7 expression, cells were treated with vehicle or specific inhibitor for 0.1 *μ*mol/l of TSA or 1 mmol/l of VPA for 24 h. Cells were harvested and RNA was used for HDAC2 expression studies by real-time PCR. To determine the effect of BMP-7 knockdown on TSA-induced suppression of cisplatin-mediated epithelial cell apoptosis, siRNA specific to BMP-7 was transfected (50 nmol/l). Twenty-four hours after transfection, cells were treated with/without cisplatin and TSA or VPA for 24 h, and then cells were harvested to quantify apoptosis by flow cytometry. At 80% confluency, cells were treated with cisplatin with/without 0.1 *μ*g/ml of BMP-7 or 0.1 *μ*mol/l of TSA or 1 mmol/l of VPA for 24 h. Cells and supernatants were harvested and subjected to cytokine and gene expression analysis.

### Quantification of apoptosis by flow cytometry

To quantify the dead cells in culture, HK-2 and mTEC cells were harvested at 24 h after cisplatin treatment (5 *μ*mol/l, 24h) with/without TSA or VPA or BMP-7 as described above. Cells were then washed and stained using Annexin-V-FITC Apoptosis Detection Kit (BestBio, Shanghai, China); samples from different groups were collected by trypsinization, and washed twice with cold phosphate-buffered saline (PBS) buffer. Before analyses were performed on a BD LSR flow cytometer (BD Biosciences, San Jose, CA, USA), cells were resuspended in 400 *μ*l Annexin-V binding buffer, added with 5 *μ*l Annexin-V-FITC cultured at 2–8 °C for 15 min, and then added with 10 *μ*l propidium iodide (PI) cultured at 2–8 °C for 5 min in dark.

### siRNA silencing

HK-2 and mTEC cells were transfected with 100 nM of siRNA using Lipofectamine 2000 (Invitrogen, Carlsbad, CA, USA) according to the manufacturer’s instructions. The oligonucleotide sequences were as follows: BMP-7 siRNA (h), 5′-CGGAAGUUCCUGUAAUAA AdTdT-3′ for the sense strand and 5′-UUUAUU ACAGGAACUUCCGdGdG-3′ for the antisense strand; HDAC2-siRNA (m), 5′-CUCAUAACUUGCUGCUAAATT-3′ for the sense strand and 5′-UUUAGCAGCAAGUUAUGAGTT-3′ for the antisense strand; HDAC2-siRNA (h), 5′-CCAGAACACUCCAGAAUAUTT-3′ for the sense strand and 5′-AUAUUCUGGAGUGUUCUGGTT-3′ for the antisense strand. A negative scrambled siRNA (Sangon Biotech, Shanghai, China) was used in parallel. Cells were cultured at 37 °C for 6 h, apoptotic cells were quantified by flow cytometry for 24h, and then western blot was used 48 h after siRNA transfection.

### Chromatin immunoprecipitation

ChIP assays were performed according to the protocol from Upstate Cell Signaling Solutions. Details of antibodies and PCR primers are as follows. Briefly, 10 million HK-2 cells were treated with 1% formaldehyde for 15 min, and the reaction was then stopped with glycine. Cells were lysed with lysis buffer with protease inhibitors. After brief centrifugation, DNA was sheared by sonication and then centrifuged again to remove cell debris. Immunoprecipitation was performed on the lysate with 4 *μ*g of anti-HDAC2 (Santa Cruz Biotechnology, Dallas, TX, USA) or anti-IgG Ab (Santa Cruz Biotechnology, Dallas, TX, USA). After washing, crosslinks were reversed with proteinase K addition. The immunoprecipitated DNA was purified using the DNA purification column from the kit and analyzed by PCR using the forward primer 5′-TCTGAGTGGTCTGGGGACTC-3′ and the reverse primer 5′- GTTCTTCCCACCT CCTCCTC-3′ specific for the human BMP-7 proximal promoter region. Input DNA was normalizad to amplification of human GAPDH promoter primer (forward: 5′-TACTAGCGGTTTTACGGGCG-3′ reverse: 5′- TCGAACAGGAGGAGCAGAGAGAGCGA-3′).

### Cr and BUN assay kits

The concentrations of Cr and BUN in serum from C57BL/6 mice with the AKI were determined by Cr and BUN assay kits according to the manufacturer’s instructions.

### Histopathology

Renal tissues of mice were fixed in 4% paraformaldehyde for 24 h immediately following killing, processed for histological examination according to a conventional method and stained with H&E. Ten fields of × 40 original magnification were examined and averaged. The slides were scored in a blinded manner and de-identified. To localize the BMP-7 expression in the kidney, sections were incubated with goat anti-BMP-7 antibody, which was followed by a secondary antibody conjugated with PI fluorescent tag.

### Immunofluorescence staining

Renal tissues of mice were fixed in 4% paraformaldehyde for 24 h immediately following killing, and staining was performed with rabbit anti-KIM-1 and goat anti-BMP-7. Similarly, cultured HK-2 and mTEC cells were induced by CP(5 *μ*mol/l, 24h), 0.1 *μ*mol/l of TSA or 1 mmol/l of VPA were used in experiments, and then cells were fixed with acetone. Staining was performed with goat anti-BMP-7. Counterstaining of secondary antibody and nuclei was performed with 4′,6-diamidino-2-phenylindole (DAPI; Beyotime Biotechnology, Shanghai, China).

### Real-time reverse transcriptase-PCR

Total RNA was collected from HK-2 and mTEC cells using TRIzol reagents (Invitrogen). First-strand cDNA was synthesized using Thermoscript RT-PCR synthesis kit (Fermentas, Pittsburgh, PA, USA) according to the manufacturer’s instructions. Real-time quantitative PCR analyses for mRNA were performed by using thermoscript RT-qPCR kits (Fermentas, Pittsburgh, PA, USA) in an ABI Prizm step-one plus real-time PCR System (Applied Biosystems, Foster City, CA, USA). The products were used as templates for amplification using the SYBR Green PCR amplification reagent (Qiagen, Valencia, CA, USA) and gene-specific primers. The primer sets used are listed in Supplementary Information.

### Western blot

Whole extracts were separated by 10 or 12% sodium dodecyl sulfate polyacrylamide gel electrophoresis (SDS-PAGE), transferred to a polyvinylidene difluoride membrane, which were incubated with primary antibodies against BMP-7 and KIM-1 (1:500; Santa Cruz biotechnology, Dallas, TX, USA), Bcl-2 and Bax (1:200; Bioss, Beijing, China). The membranes were then washed in TBS/Tween 20 and incubated with secondary antibodies correspondingly. After extensive washing in TBS/Tween 20, protein bands were visualized with ECL-chemiluminescent kit (ECL-plus; Thermo Fisher Scientific, Pittsburgh, PA, USA).

### Assay of caspase-3 activity

The activity of caspase-3 was measured by using the caspase-3 activity kit (Bestbio, Shanghai, China), according to the manufacturer’s instruction. Assays were performed on 96-well microtiter plates. Ten microliters of protein extracts, 90 *μ*l of reaction buffer and 10 *μ*l of caspase substrate were added by turns. Then, the protein extracts were incubated at 37 °C for 2–3 h. Samples were measured with Multiskan MK3 (Biotek, Winooski, VT, USA) at an absorbance of 405 nm.

### Assay of HDAC2 activity

HDAC2 activity was measured using a colorimetric assay kit (Genmed, Wilmington, DE, USA). Briefly, nuclear extract samples were incubated with HDAC2 colorimetric substrate (Boc-Lys (Ac)-pNA) with specific inhibitors (apicidin). The deacetylation sensitizes the substrate, so subsequent treatment with the aminopeptidase produces a fluorophore that can then be measured using a fluorescence reader. HDAC2 activity was expressed as relative OD values.

### TUNEL assay

Tumor apoptosis was measured using a TUNEL assay (Kegen biothech, Nanjing, China). The renal cryostat sections (7 mm) were prepared and perfused in 4% paraformaldehyde for 30 min. After permeabilized with 1% Triton X-100 for 5 min, incubated with 50 *μ*l of TDT and Streptavidin-Fluorescein at 37 °C for 30 min, nuclei were performed with DAPI (Beyotime Biotechnology, Shanghai, China).

### Statistical analysis

Data are represented as mean±S.D. Statistical analysis was performed using ANOVA followed by Student’s *t*-test. For changes in mRNA or protein levels, ratios of mRNA (relative expression) and protein (densitometric values) to respective house-keeping controls were compared. Significance was defined as *P*<0.05.
