# Supplementary material for: Suppression of BMP-7 by histone deacetylase 2 promoted apoptosis of renal tubular epithelial cells in acute kidney injury
Source: Cell Death Dis. 2017 Oct 26;8(10):e3139–. doi: 10.1038/cddis.2017.552 (PMC5680919; doi:10.1038/cddis.2017.552)
Supplement: Supplementary Figures [file cddis2017552x1.ppt]

## Slide 1
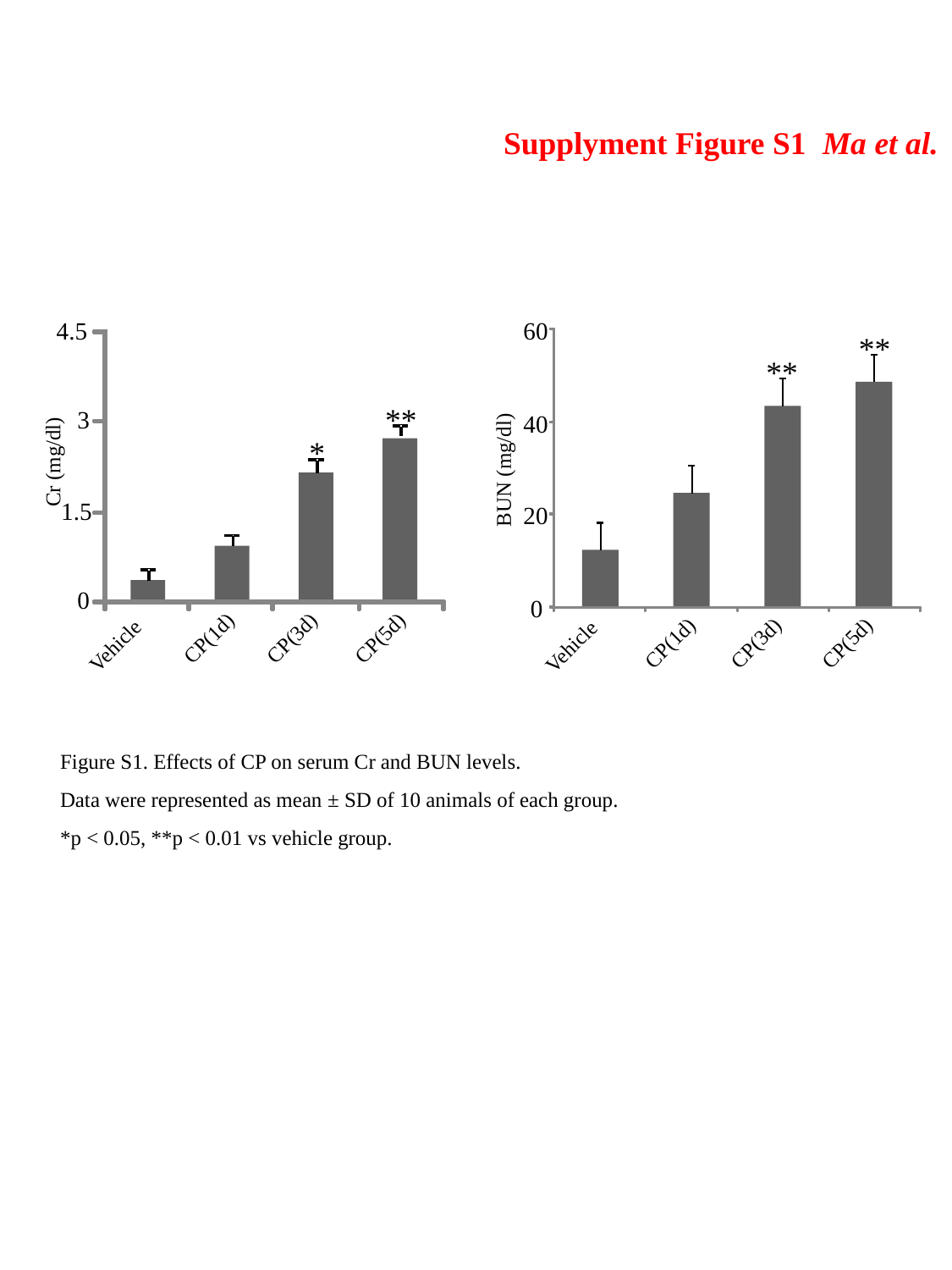

Supplyment Figure S1 Ma et al.
4.5
3
1.5
0
Cr (mg/dl)
**
*
Vehicle
CP(1d)
CP(3d)
CP(5d)
60
40
20
0
**
BUN (mg/dl)
**
Vehicle
CP(1d)
CP(3d)
CP(5d)
Figure S1. Effects of CP on serum Cr and BUN levels.
Data were represented as mean ± SD of 10 animals of each group.
*p < 0.05, **p < 0.01 vs vehicle group.

## Slide 2
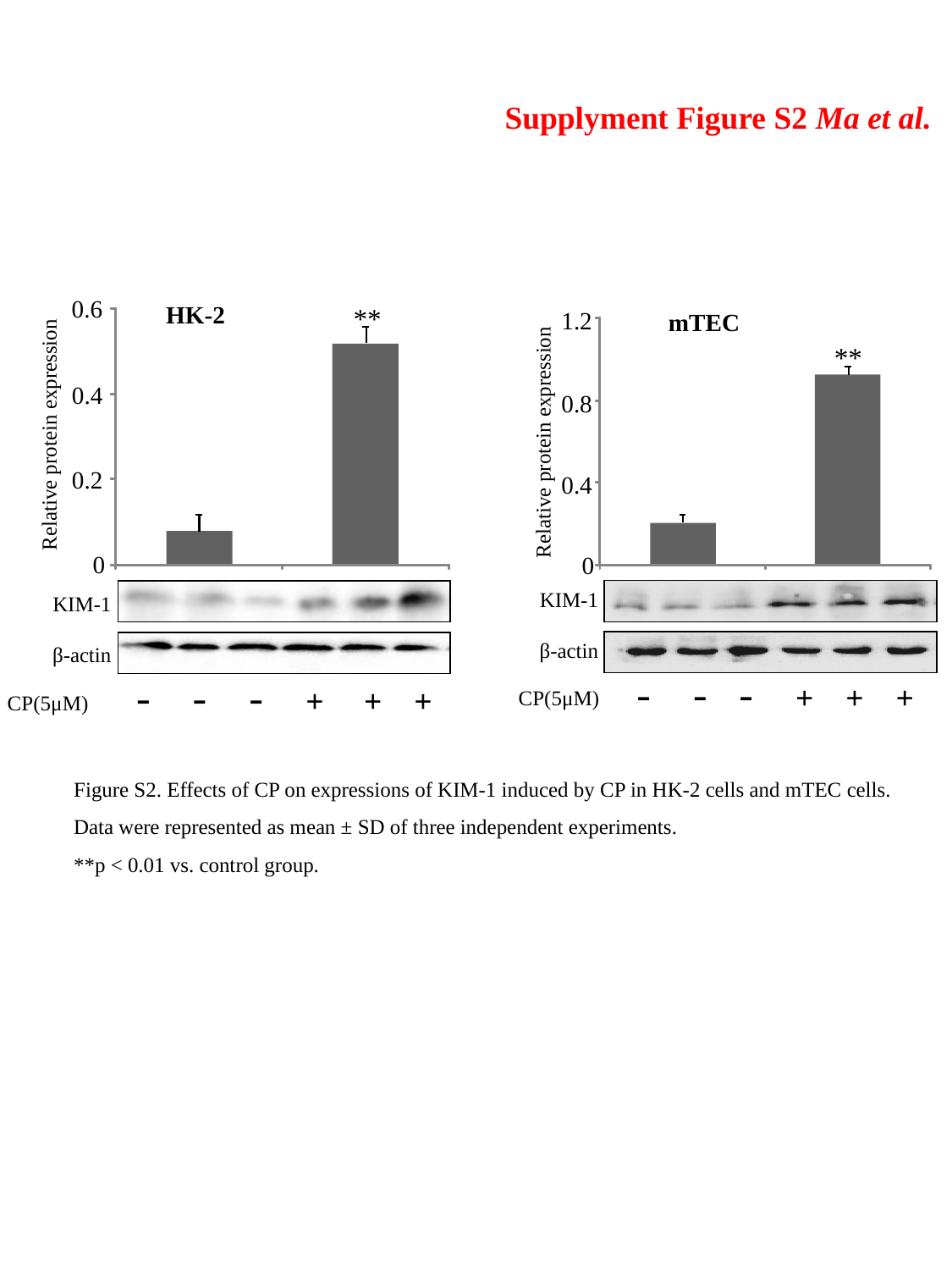

Supplyment Figure S2 Ma et al.
Relative protein expression
0.6
0.4
0.2
0
**
HK-2
 - - - + + +
CP(5μM)
Relative protein expression
mTEC
1.2
0.8
0.4
0
**
KIM-1
β-actin
KIM-1
β-actin
- - - + + +
CP(5μM)
Figure S2. Effects of CP on expressions of KIM-1 induced by CP in HK-2 cells and mTEC cells.
Data were represented as mean ± SD of three independent experiments.
**p < 0.01 vs. control group.

## Slide 3
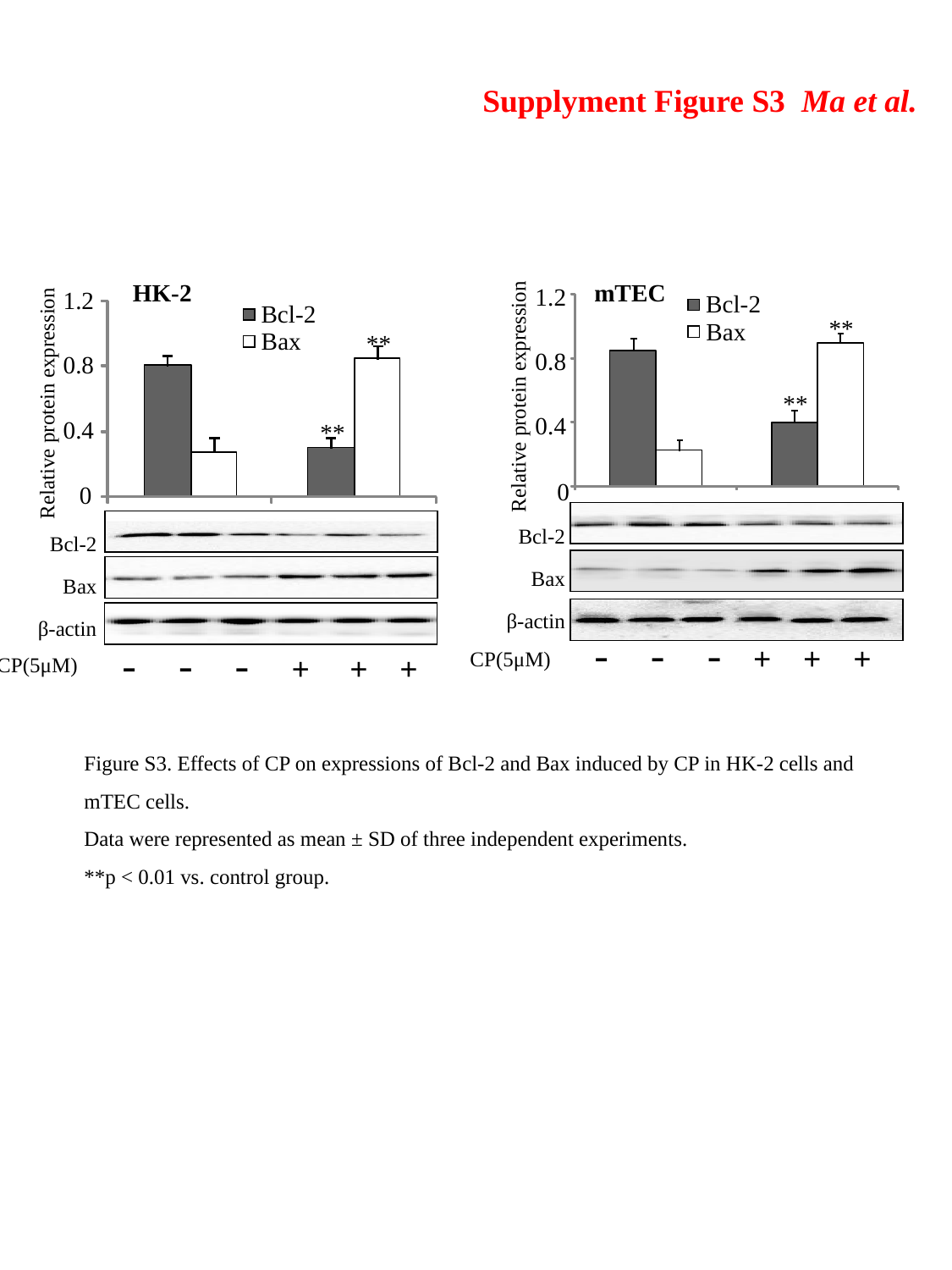

Supplyment Figure S3 Ma et al.
Relative protein expression
mTEC
1.2
0.8
0.4
0
Bcl-2
Bax
Bcl-2
Bax
β-actin
 - - - + + +
CP(5μM)
Relative protein expression
HK-2
1.2
Bcl-2
Bax
0.8
0.4
0
Bcl-2
Bax
β-actin
 - - - + + +
CP(5μM)
**
**
**
**
Figure S3. Effects of CP on expressions of Bcl-2 and Bax induced by CP in HK-2 cells and mTEC cells.
Data were represented as mean ± SD of three independent experiments.
**p < 0.01 vs. control group.

## Slide 4
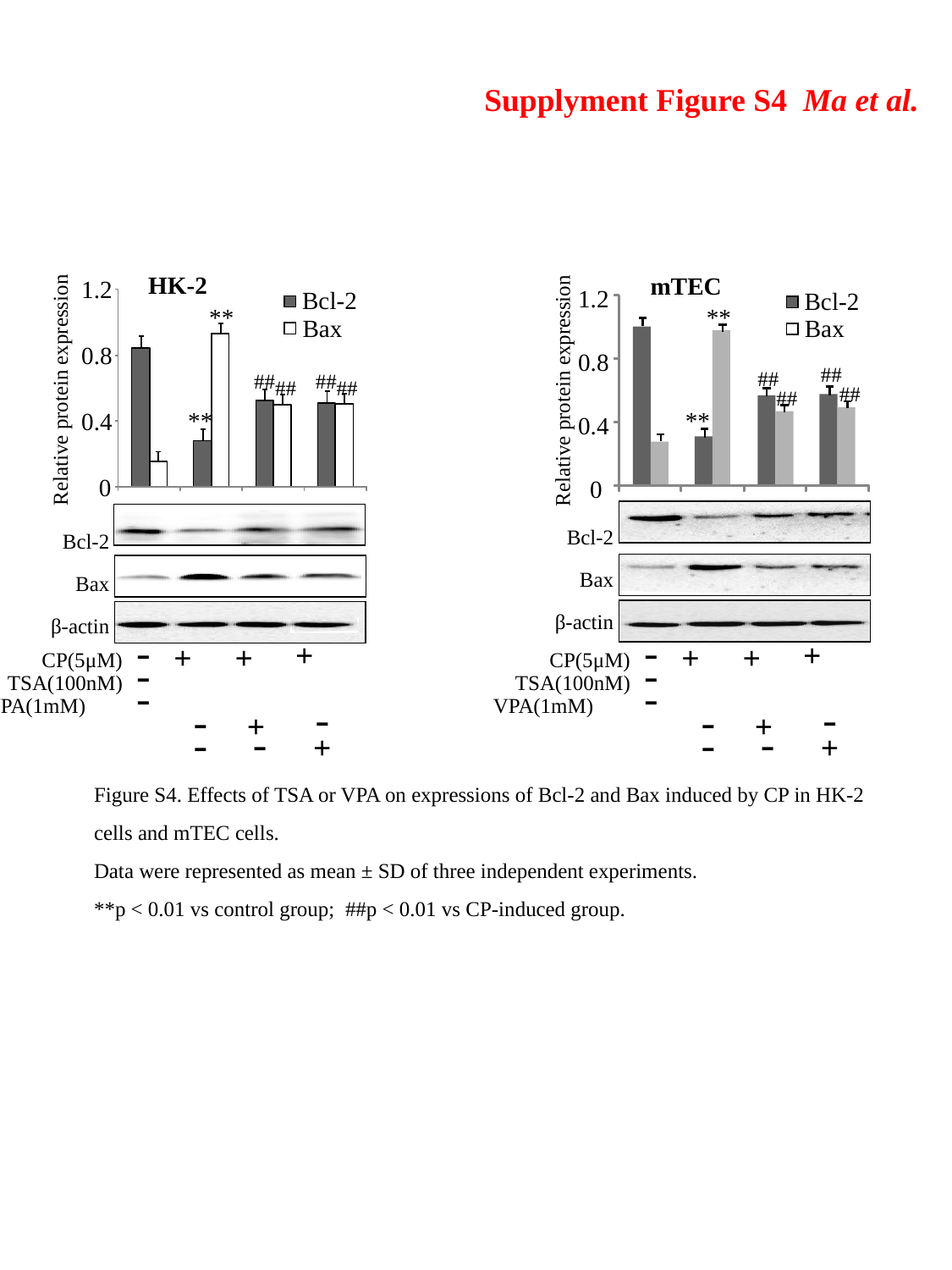

Supplyment Figure S4 Ma et al.
Relative protein expression
HK-2
1.2
0.8
0.4
0
Bcl-2
**
Bax
##
##
##
##
**
Bcl-2
Bax
β-actin
CP(5μM)
TSA(100nM)
VPA(1mM)
+
-
+
+
+
-
-
-
-
+
-
-
Relative protein expression
mTEC
1.2
0.8
0.4
0
Bcl-2
**
Bax
##
##
##
##
**
Bcl-2
Bax
β-actin
CP(5μM)
TSA(100nM)
VPA(1mM)
+
-
+
+
+
-
-
-
-
+
-
-
Figure S4. Effects of TSA or VPA on expressions of Bcl-2 and Bax induced by CP in HK-2 cells and mTEC cells.
Data were represented as mean ± SD of three independent experiments.
**p < 0.01 vs control group; ##p < 0.01 vs CP-induced group.

## Slide 5
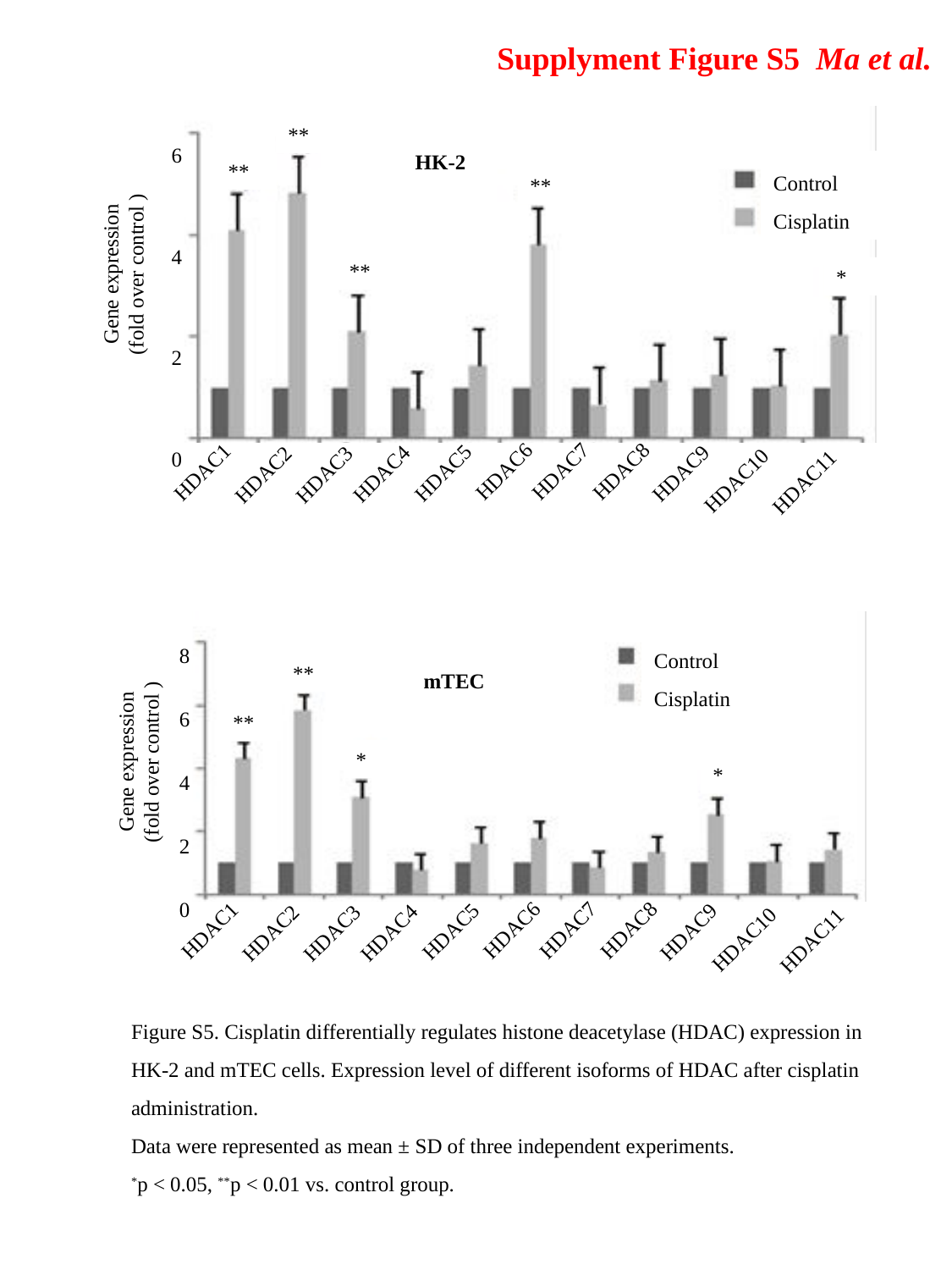

Supplyment Figure S5 Ma et al.
6
4
2
0
HK-2
mTEC
**
Control
Cisplatin
**
**
Gene expression
(fold over control )
**
*
HDAC6
HDAC7
HDAC8
HDAC1
HDAC5
HDAC9
HDAC4
HDAC2
HDAC3
HDAC11
HDAC10
8
6
4
2
0
Control
Cisplatin
**
**
Gene expression
(fold over control )
*
*
HDAC6
HDAC7
HDAC8
HDAC1
HDAC5
HDAC9
HDAC4
HDAC2
HDAC3
HDAC11
HDAC10
Figure S5. Cisplatin differentially regulates histone deacetylase (HDAC) expression in HK-2 and mTEC cells. Expression level of different isoforms of HDAC after cisplatin administration.
Data were represented as mean ± SD of three independent experiments.
*p < 0.05, **p < 0.01 vs. control group.

## Slide 6
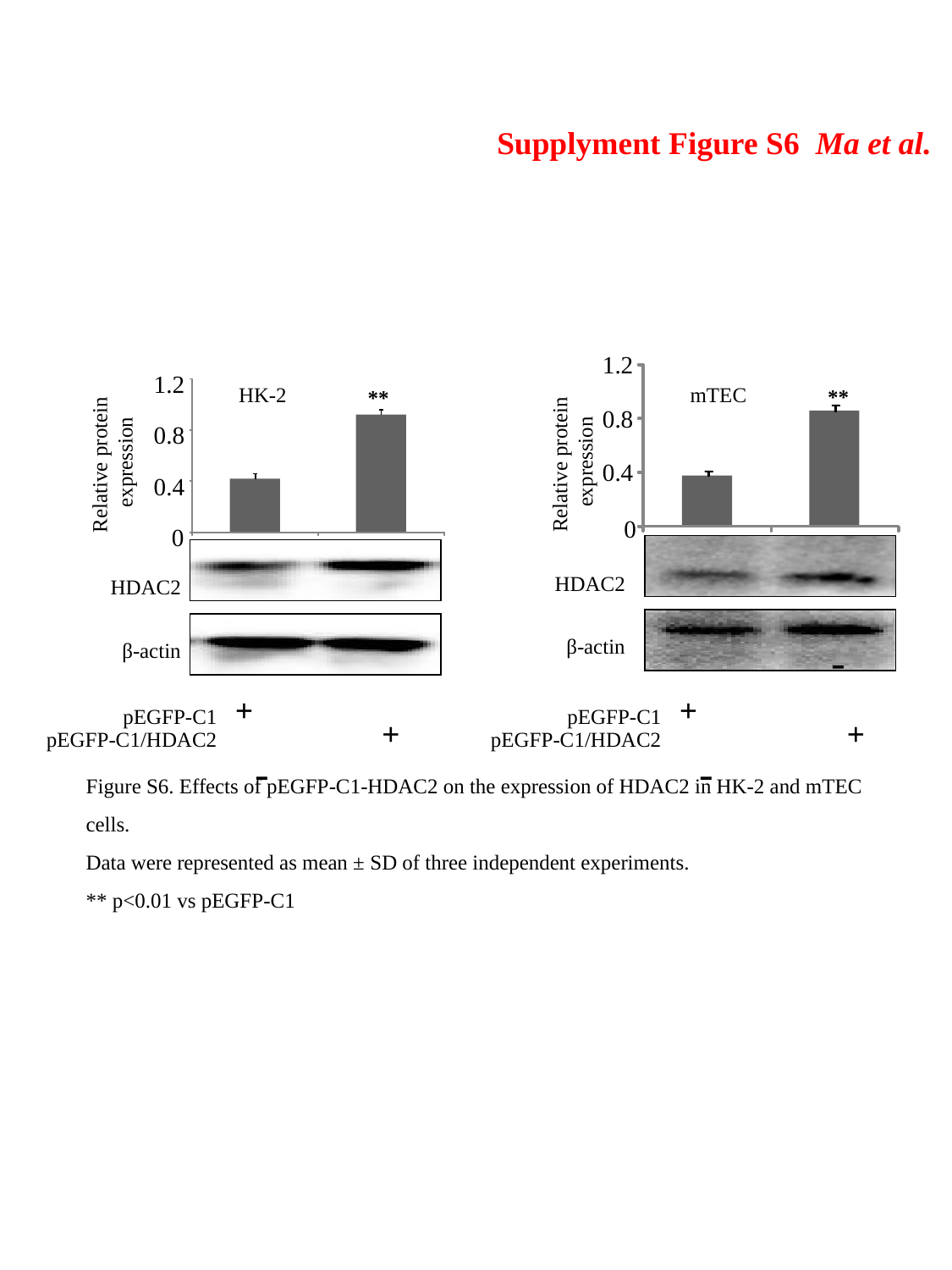

Supplyment Figure S6 Ma et al.
1.2
0.8
0.4
0
mTEC
Relative protein
expression
**
HDAC2
β-actin
+
-
pEGFP-C1
pEGFP-C1/HDAC2
+
-
HK-2
Relative protein
expression
1.2
0.8
0.4
0
**
HDAC2
β-actin
+
-
pEGFP-C1
pEGFP-C1/HDAC2
+
-
Figure S6. Effects of pEGFP-C1-HDAC2 on the expression of HDAC2 in HK-2 and mTEC cells.
Data were represented as mean ± SD of three independent experiments.
** p<0.01 vs pEGFP-C1

## Slide 7
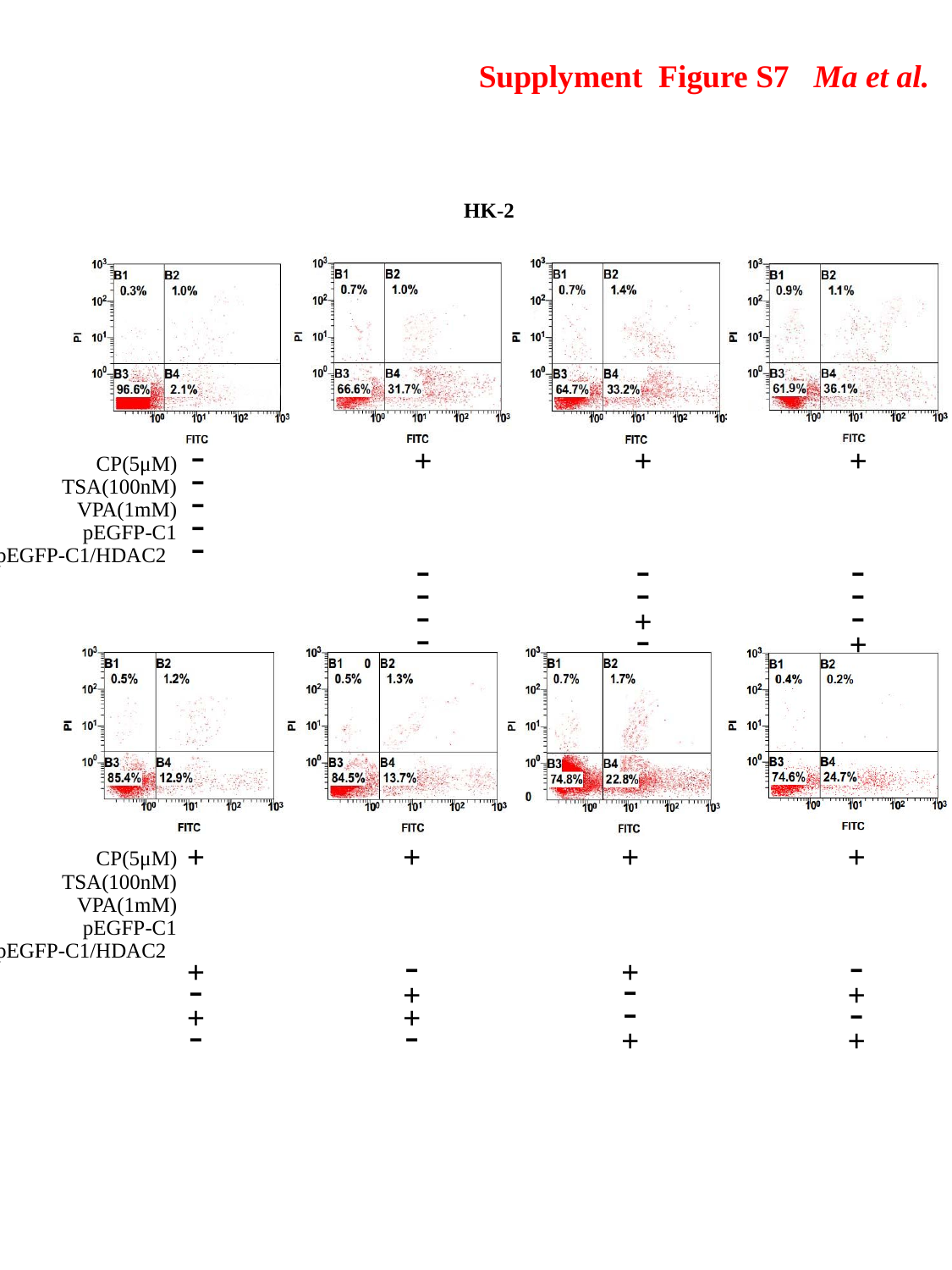

Supplyment Figure S7 Ma et al.
HK-2
CP(5μM)
TSA(100nM)
VPA(1mM)
pEGFP-C1
pEGFP-C1/HDAC2
+
-
-
-
-
-
-
-
-
-
+ -
-
+
-
+ -
-
-
+
CP(5μM)
TSA(100nM)
VPA(1mM)
pEGFP-C1
pEGFP-C1/HDAC2
+
+
-
-
+
+ +
-
+
-
+
-
+
+
-
+
-
+
-
+

## Slide 8
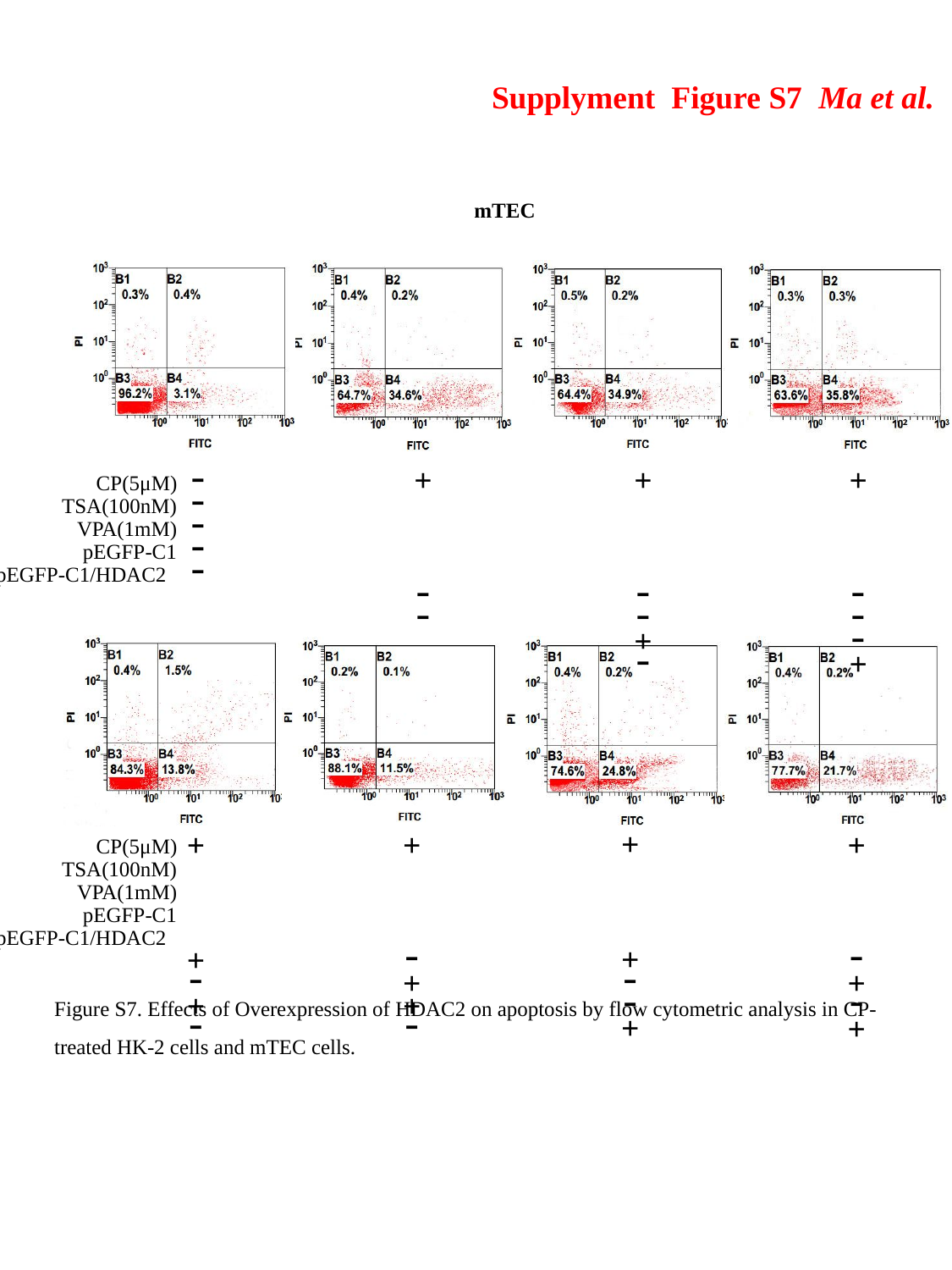

Supplyment Figure S7 Ma et al.
mTEC
CP(5μM)
TSA(100nM)
VPA(1mM)
pEGFP-C1
pEGFP-C1/HDAC2
+
-
-
-
-
-
-
-
-
-
+ -
-
+
-
+ -
-
-
+
CP(5μM)
TSA(100nM)
VPA(1mM)
pEGFP-C1
pEGFP-C1/HDAC2
+
+
-
-
+
+ +
-
+
-
+
-
+
+
-
+
-
+
-
+
Figure S7. Effects of Overexpression of HDAC2 on apoptosis by flow cytometric analysis in CP-treated HK-2 cells and mTEC cells.

## Slide 9
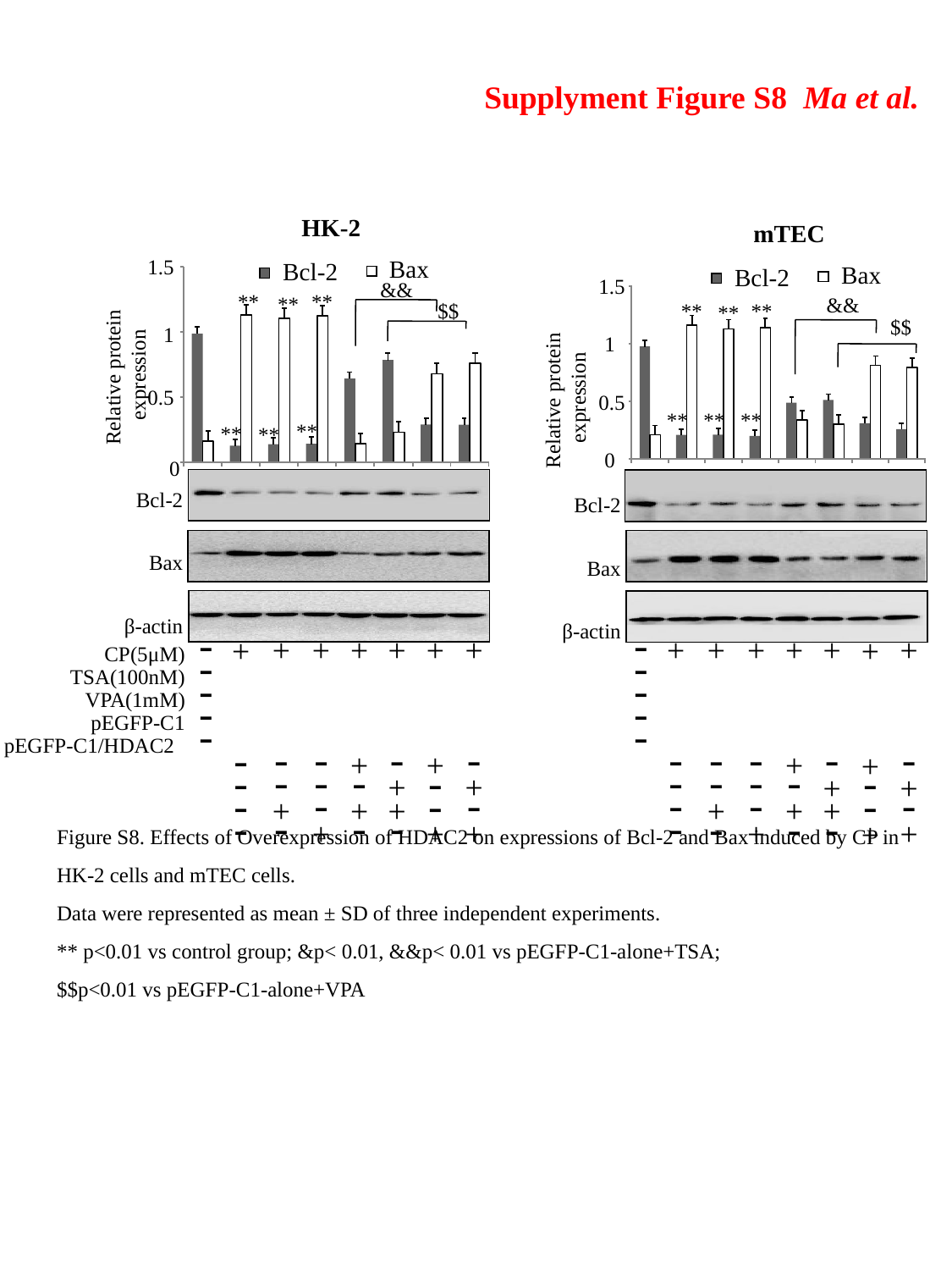

Supplyment Figure S8 Ma et al.
HK-2
Bax
Bcl-2
mTEC
1.5
1
0.5
0
Bax
Bcl-2
Relative protein
expression
&&
**
**
**
$$
**
**
1.5
1
0.5
0
&&
**
**
**
$$
**
**
**
Relative protein
expression
**
Bcl-2
Bax
β-actin
Bcl-2
Bax
β-actin
CP(5μM)
TSA(100nM)
VPA(1mM)
pEGFP-C1
pEGFP-C1/HDAC2
-
-
-
-
-
+ -
-
+
-
+ -
-
-
+
+ +
-
+
-
+
-
+
+
-
+
-
+
-
+
+
-
-
-
-
-
-
-
-
-
+ -
-
+
-
+ -
-
-
+
+ +
-
+
-
+
-
+
+
-
+
-
+
-
+
+
+
-
-
+
+
-
-
-
-
+
+
-
-
+
Figure S8. Effects of Overexpression of HDAC2 on expressions of Bcl-2 and Bax induced by CP in HK-2 cells and mTEC cells.
Data were represented as mean ± SD of three independent experiments.
** p<0.01 vs control group; &p< 0.01, &&p< 0.01 vs pEGFP-C1-alone+TSA;
$$p<0.01 vs pEGFP-C1-alone+VPA

## Slide 10
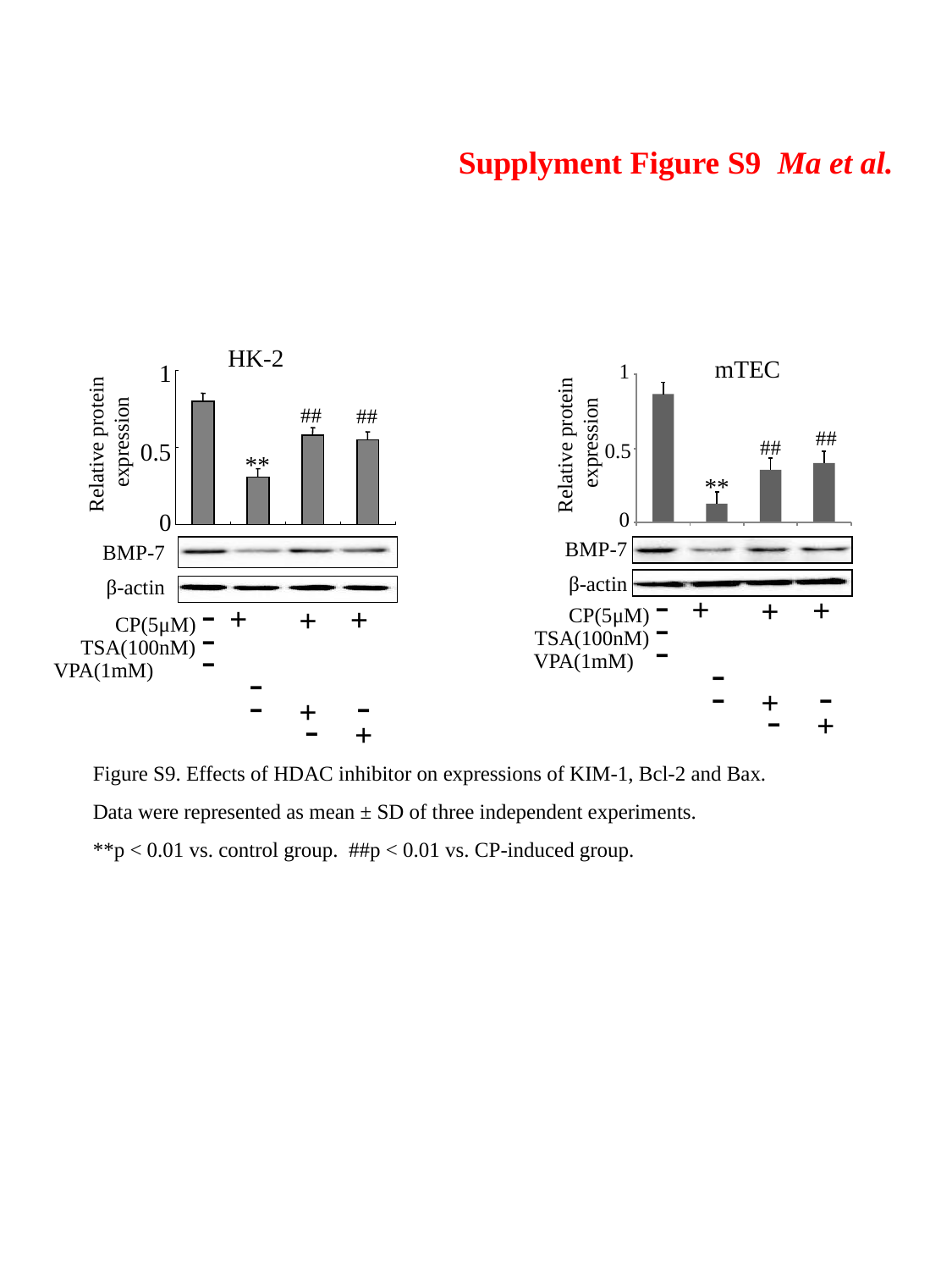

Supplyment Figure S9 Ma et al.
Relative protein
expression
HK-2
Relative protein
expression
mTEC
1
0.5
0
##
##
**
BMP-7
β-actin
CP(5μM)
TSA(100nM)
VPA(1mM)
+
-
-
+
-
+
-
-
-
+
+
-
1
0.5
0
##
##
**
BMP-7
β-actin
CP(5μM)
TSA(100nM)
VPA(1mM)
+
-
-
+
-
+
-
-
-
+
+
-
Figure S9. Effects of HDAC inhibitor on expressions of KIM-1, Bcl-2 and Bax.
Data were represented as mean ± SD of three independent experiments.
**p < 0.01 vs. control group. ##p < 0.01 vs. CP-induced group.

## Slide 11
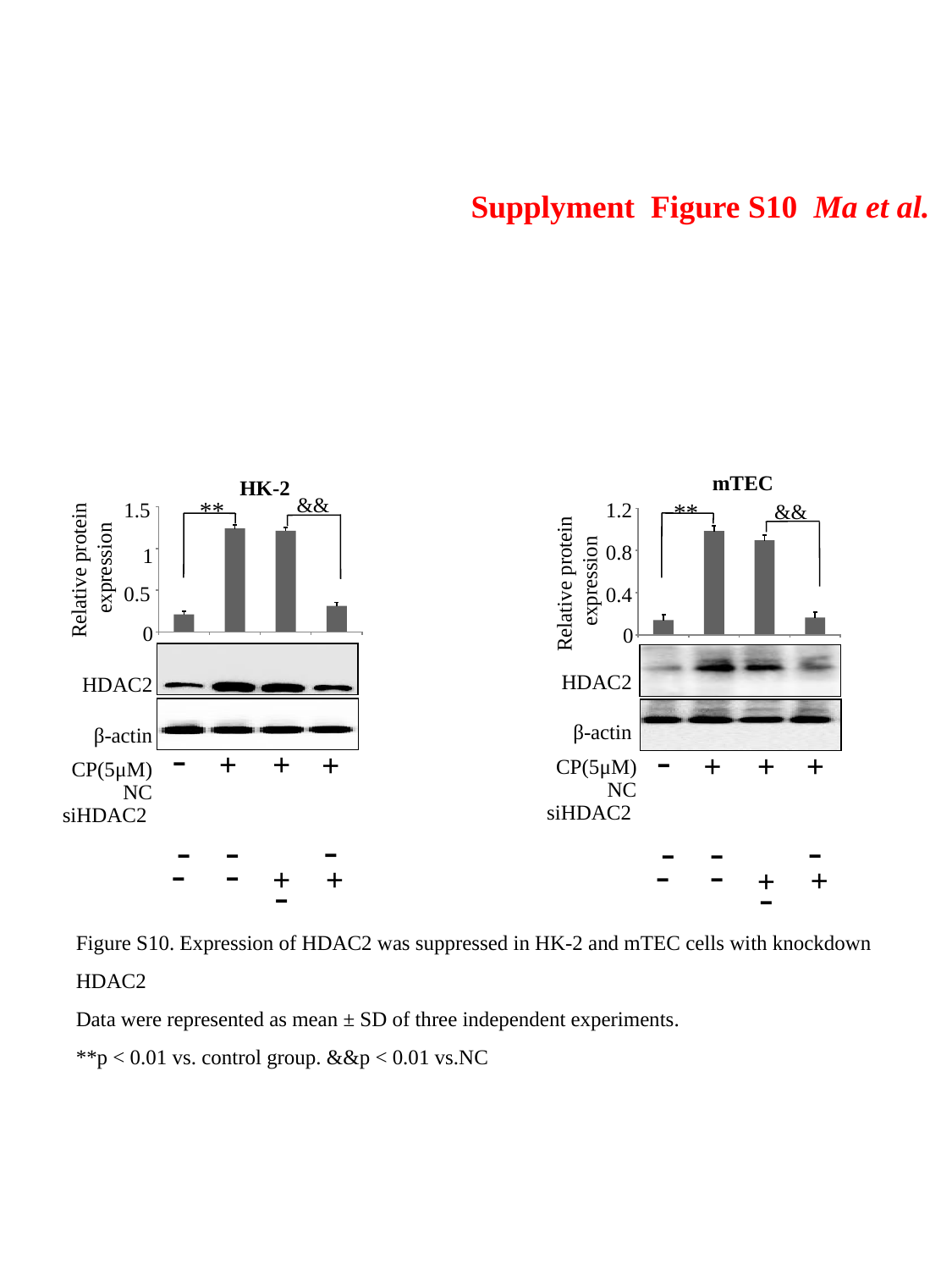

Supplyment Figure S10 Ma et al.
mTEC
HK-2
Relative protein
 expression
Relative protein
 expression
&&
**
**
&&
1.5
1
0.5
0
1.2
0.8
0.4
0
HDAC2
β-actin
HDAC2
β-actin
CP(5μM)
NC
siHDAC2
CP(5μM)
NC
siHDAC2
+
+
-
+
-
+
-
-
-
+
-
-
+
-
+
-
-
-
+
-
-
+
+
-
Figure S10. Expression of HDAC2 was suppressed in HK-2 and mTEC cells with knockdown HDAC2
Data were represented as mean ± SD of three independent experiments.
**p < 0.01 vs. control group. &&p < 0.01 vs.NC

## Slide 12
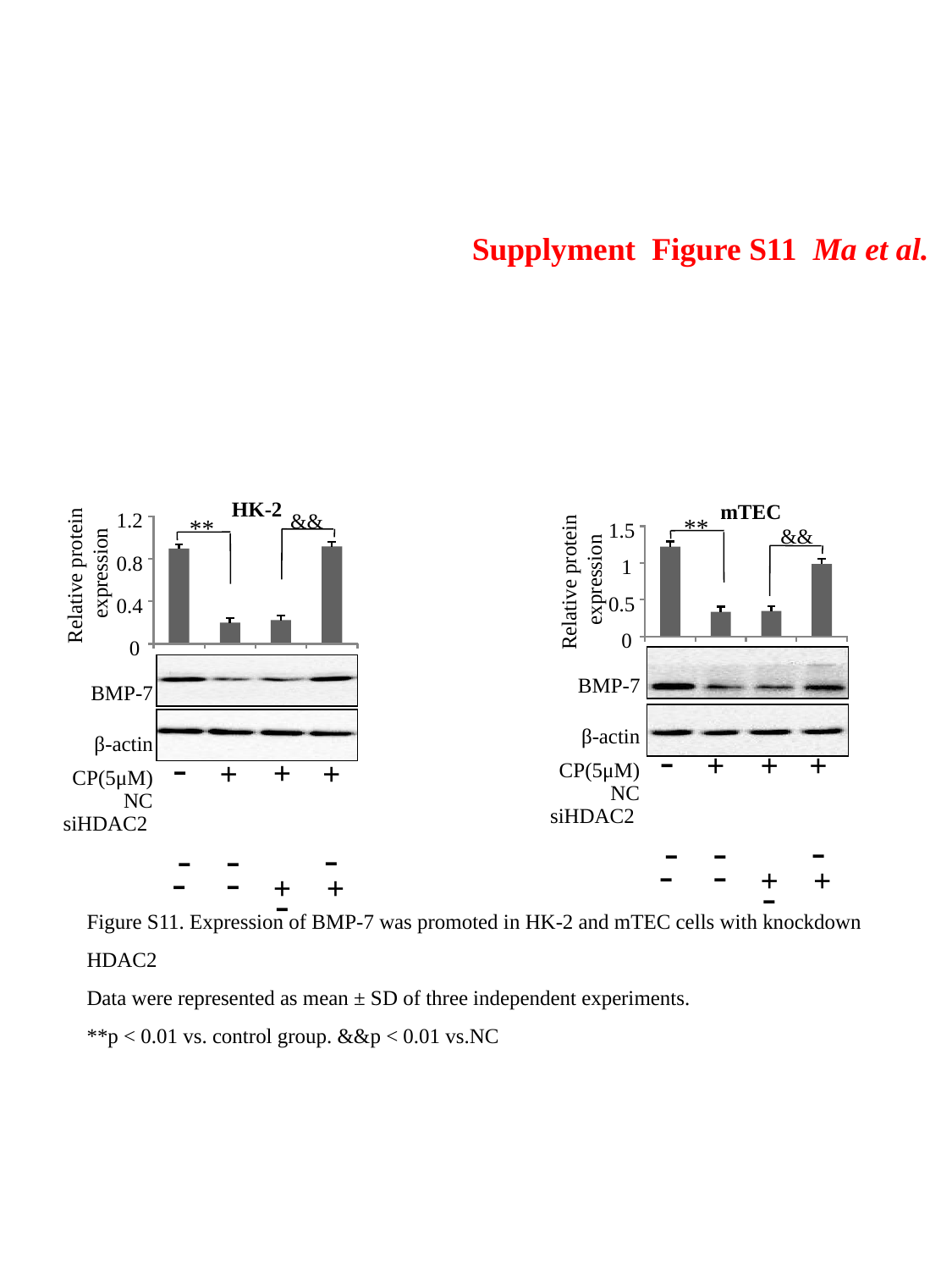

Supplyment Figure S11 Ma et al.
HK-2
mTEC
Relative protein
 expression
&&
**
Relative protein
 expression
**
1.2
0.8
0.4
0
&&
1.5
1
0.5
0
BMP-7
β-actin
BMP-7
β-actin
CP(5μM)
NC
siHDAC2
+
+
-
+
-
+
-
-
-
+
-
-
CP(5μM)
NC
siHDAC2
+
+
-
+
-
+
-
-
-
+
-
-
Figure S11. Expression of BMP-7 was promoted in HK-2 and mTEC cells with knockdown HDAC2
Data were represented as mean ± SD of three independent experiments.
**p < 0.01 vs. control group. &&p < 0.01 vs.NC

## Slide 13
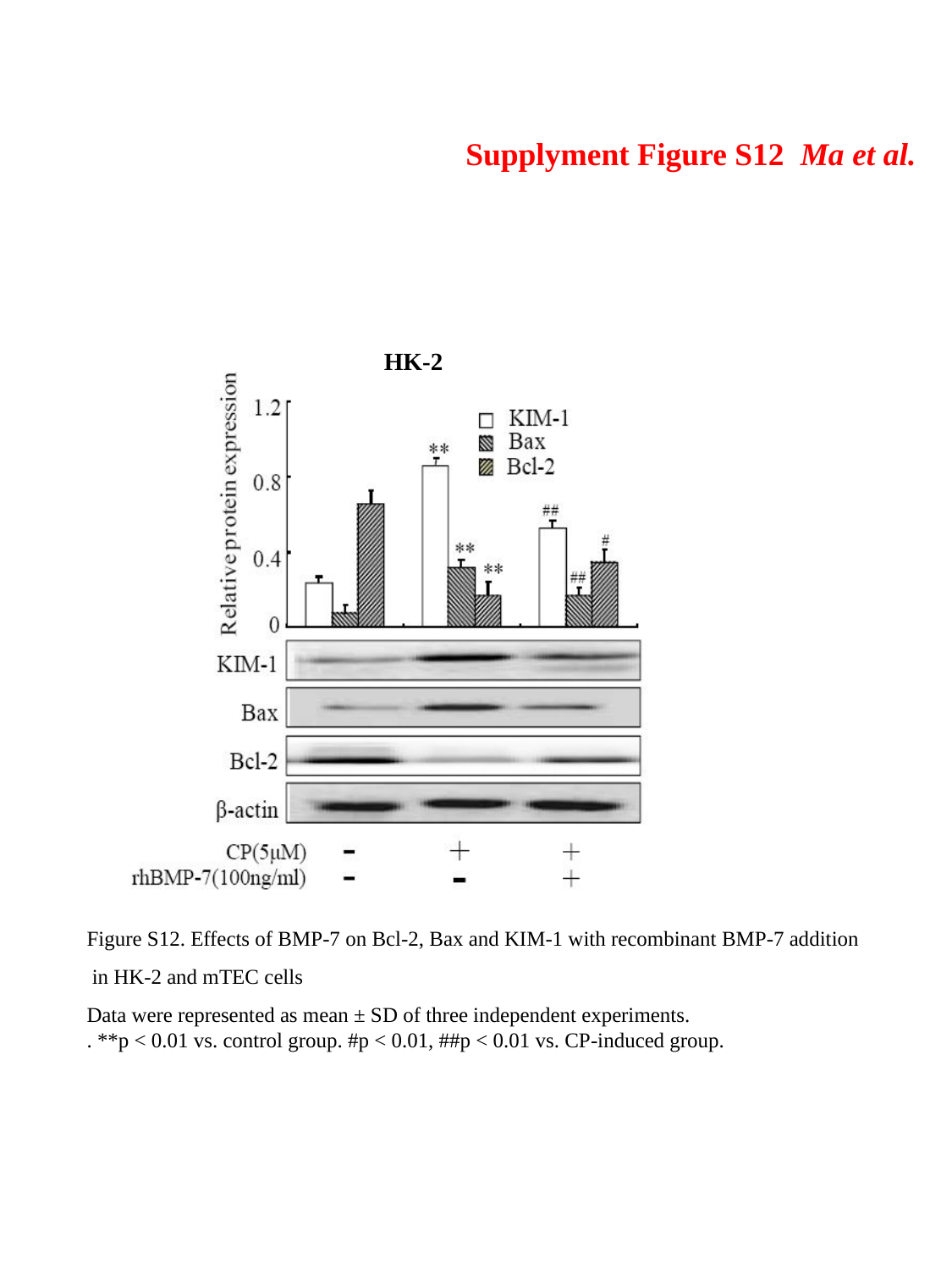

Supplyment Figure S12 Ma et al.
HK-2
Figure S12. Effects of BMP-7 on Bcl-2, Bax and KIM-1 with recombinant BMP-7 addition in HK-2 and mTEC cells
Data were represented as mean ± SD of three independent experiments.
. **p < 0.01 vs. control group. #p < 0.01, ##p < 0.01 vs. CP-induced group.

## Slide 14
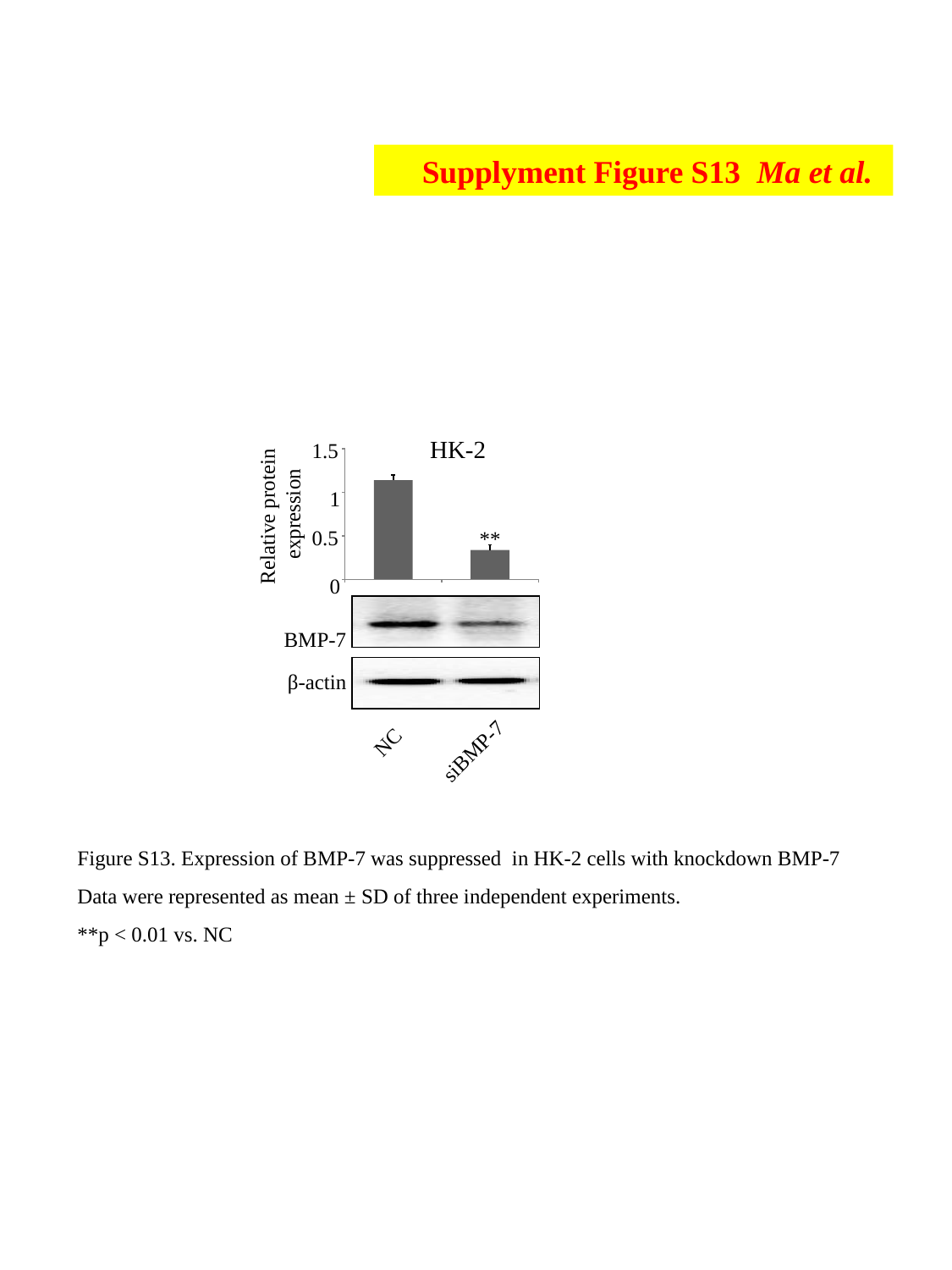

Supplyment Figure S13 Ma et al.
Relative protein
expression
1.5
1
0.5
0
**
BMP-7
β-actin
NC
siBMP-7
HK-2
Figure S13. Expression of BMP-7 was suppressed in HK-2 cells with knockdown BMP-7
Data were represented as mean ± SD of three independent experiments.
**p < 0.01 vs. NC
