# Supplementary material for: Suppression of BMP-7 by histone deacetylase 2 promoted apoptosis of renal tubular epithelial cells in acute kidney injury
Source: Cell Death Dis. 2017 Oct 26;8(10):e3139–. doi: 10.1038/cddis.2017.552 (PMC5680919; doi:10.1038/cddis.2017.552)
Supplement: Supplementary Information [file cddis2017552x2.pdf]

Supplyment information

The primer sets

name	Forward primer	Reverse primer	NCBI Reference Sequence
mouse HDAC1	5′GACCCTGACAAAC GCATCTC3′	5′GTTCTTGCGACCAC CTTCTC3′	NM_008228.2
mouse HDAC2	5′CACATGCACCTGGT GTTCAA3′	5′TCGCAAGCTATCCG TTTGTC3′	NM_008229.2
mouse HDAC3	5′AGTTCTGCTCCCGT TACACA3′	5′AGAAGCCAGAGGC CTCAAAT3′	NM_010411.2
mouse HDAC4	5′GAAACGAGCTTGA GCCTCTG3′	5′GCCAGTACTTGCTG TGGATG3′	NM_207225.2
mouse HDAC5	5′GTGGCAGTGGAGGT GAAGCC3′	5′GCAGGGTCCATGGG GCCCGC3′	NM_001284248.1
mouse HDAC6	5′CCAGCCTCGCATAC AAACAA3′	5′ATCATGGGCTTCCT CTGCTT3′	NM_001130416.1
mouse HDAC7	5′GGACGTTTGTGATG CTACCC3′	5′CGGGAAGCTACTTT GAAGGC3′	NM_001204281.1
mouse HDAC8	5′TGCAGCAGCTATAG GAGGAG3′	5′ACCCTCCAGACCAG TTGATG3′	NM_027382.4
mouse HDAC9	5′ACAACTCCAGGAGC ACATCA3′	5′GCGATGCCTCTCTA CTTCCT3′	NM_001271386.1
mouse HDAC10	5′TCCAGGATGAGGAT CTTGCC3′	5′GCTAGGCATCGCTG AATGAG3′	NR_028449.1
mouse HDAC11	5′AAGAGAAGCTGCT GTCCGAT3′	5′ACCACTTCAGCTCG TTGAGA3′	NM_144919.2
Human HDAC1	5′ATTATGGACAAGGC CACCCA3′	5′TCATCGCTGTGGTA CTTGGT3′	NM_004964.2
Human HDAC2	5′GATGTTCTGGCATC CTCCCT3′	5′TGTTCCATCTCCTCC ATCCAC3′	NM_001527.3
Human HDAC3	5′CATCTCTGCAAGGA GCAACC3′	5′GAGGCCTCAAACTT CTTGGC3′	NM_003883.3
Human HDAC4	5′CAAAGACCCAATGC AAACGC3′	5′GCTTCTTCGTTCTC GCAAGT3′	NM_006037.3
Human HDAC5	5′TACAGCCTTCAGGA CAGTGG3′	5′ATCTGGCGGTGACA GAGTAG3′	NM_001015053.1
Human HDAC6	5′CCAGGCTTCAGTTT 5′CCTGTG3′	5′TGTCCTCCTCCATG TTGTCC3′	NM_001321229.1
Human HDAC7	5′TGAGTACCTGGCTG CTTTCA3′	5′GCAGCATCAAATCC AGCAGA3′	NM_001308090.1
Human HDAC8	5′CTCCAGAAGGTCAG CCAAGA3′	5′GGCAGCTGTGATCG TAGCCC3′	NM_018486.2
Human HDAC9	5′CCATCCCAAGCTCT GGTACA3′	5′TGCATCTTGTGCTC CTGGTA3′	NM_058176.2
Human HDAC10	5′TGCACAGCCCAGAG TATGTA3′	5′AGTAGATGGCGTCG AACTGT3′	NM_032019.5
Human HDAC11	5′GCCCATCCTTATGGT GACCT3′	5′GGTGTGTCTGAGTT CTGTGC3′	NM_024827.3
